# Preparation of Large Conjugated Polybenzimidazole Fluorescent Materials and Their Application in Metal Ion Detection

**DOI:** 10.3390/polym13183091

**Published:** 2021-09-14

**Authors:** Xi-Ying Cao, Chu-Ming Pang, Ying Xiao, Wan-Qing Xiao, Shi-He Luo, Jin-Ping He, Zhao-Yang Wang

**Affiliations:** 1 School of Chemistry, South China Normal University, Key Laboratory of Theoretical Chemistry of Environment, Ministry of Education; Guangzhou Key Laboratory of Analytical Chemistry for Biomedicine, Guangzhou 510006, China; 2019022188@m.scnu.edu.cn (X.-Y.C.); 2019022193@m.scnu.edu.cn (Y.X.); 2020022598@m.scnu.edu.cn (W.-Q.X.); 20172421078@m.scnu.edu.cn (J.-P.H.); 2 School of Health Medicine, Guangzhou Huashang College, Guangzhou 511300, China

**Keywords:** large conjugation, polybenzimidazole, fluorescent probe, *N*-alkylation modification

## Abstract

A new type of conjugated polybenzimidazole (**CPBI**) was synthesized through a simple polycondensation reaction without metal catalysis, and *N*-alkylation modification was carried out to solve the problems of solubility and fluorescence properties. A series of nano-microsphere polymers **CPBI*_n_*** with large conjugation, good solubility, and strong fluorescence has been successfully used as “turn-off” fluorescent probes for the first time. The results show that, under suitable *N*-alkylation conditions, the obtained **CPBI*_n_*** can be used as a highly sensitive and selective fluorescent probe for the detection of Cu^2+^ and Zn^2+^ at the same time, and their detection limits are both nM levels. In addition, **CPBI_2_** can be designed as an ultra-sensitive IMPLICATION logic gate at the molecular level, cyclically detecting Cu^2+^. With the test paper containing **CPBI_2_**, easy and quick on-site detection can be achieved. This research provides a new idea for the brief synthesis of multifunctional materials.

## 1. Introduction

Conjugated polymer molecules are important materials for optoelectronic applications because they provide a relatively direct link between optoelectronic properties and compound structures [1]. Compared with non-backbone conjugated polymeric probes, organic backbone conjugated polymers are composed of unsaturated structural units, such as aromatic hydrocarbons, olefins, or acetylene, making the formation of a large delocalized polarizable π-electronic domain easy [2]. For their excellent optical and electrical properties, more and more attention has been paid to them [3]. At the same time, their molecular chains may function as “molecular lines”, along which energy and electric charges can move. This unique performance determines the ability to detect ultra-low concentrations of analytes [4,5,6]. Therefore, the synthesis and application of organic conjugated macromolecular chemical sensors have attracted more and more attention [7,8,9].

Copper ion and zinc ions are essential trace elements in the human body, which play an important role in human health, especially the catalytic auxiliary role in various biological processes such as the function of many cellular enzymes and proteins [10,11,12]. However, when the concentration of absorbed ions is unbalanced, the homeostasis in cells will be affected, leading to a series of diseases [13,14,15]. The multifunctional detection of the same sensor for each detection object is attracting attention due to its high efficiency [16,17,18,19]. As a result, the design and synthesis of new multifunctional fluorescent probes that can simultaneously detect copper and zinc ions are of great significance in biomedical and environmental monitoring applications [20,21].

According to the structural characteristics of the analyte, the design of fluorescent molecules is crucial. Specific recognition units are introduced, such as *N*-containing heterocyclic compounds such as quinoline [22], imidazole [23,24], and carbazole [25], to provide a definite binding site for metal ion as analyte. Using the availability, strong polarization, and π–π electron transition function of the conjugate system to ensure its energy transfer or electron transfer [26], the multifunctional probe may be constructed. In addition, as an effective method to achieve fluorescence probe regulation [27,28], the introduction of a long alkyl chain at the end of the compound increases the spatial effect of the polymer, significantly affecting the HOMO energy level, keeping the LUMO energy level basically unchanged, increasing the band gap [29], and affecting its optical properties, which is of great research value [30,31,32].

**Scheme 1:**
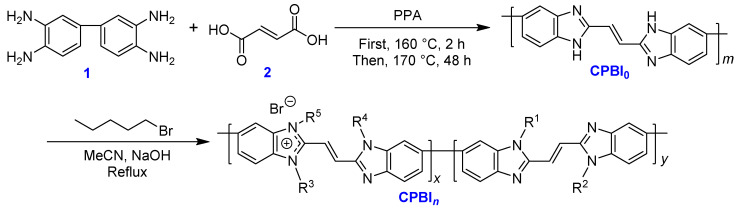
Synthesis of serial **CPBIs** fluorescent materials.

Herein, we used (*E*)-2-butenedioic acid (*E*-BA) as a “bridge” by two steps to synthesize a novel conjugated nanosphere structure polybenzimidazole sensor **CPBI*_n_*** (Scheme 1), which can be used for Cu^2+^ and Zn^2+^ detection with fluorescence regulation. Additionally, through infrared spectrum (IR), X-ray photoelectron spectroscopy (XPS), scanning electron microscope (SEM), etc., their structures and photophysical properties were studied. The results show that **CPBI*_n_*** with good solubility and optical properties can be successfully obtained by modifying **CPBI_0_** with an alkyl chain and used as a multifunctional fluorescent probe to detect Cu^2+^ and Zn^2+^ at the same time, with a low detection limit.

## 2. Materials and Methods

### 2.1. Apparatus and Reagents

All reagents and organic solvents were purchased from commercial suppliers and used without further purification. ^1^H NMR spectra of compounds were recorded on a Bruker 600 MHz instrument (Bruker AVANCE NEO, Bruker, Karlsruhe, Germany). Fluorescence spectra were carried out by a Hitachi F-4600 fluorescence spectrometer (Hitachi, Tokyo, Japan). The Scanning Electron Microscope was obtained by FEI Quanta 250 FEG field emission scanning electron microscope (FEI Quanta 250 FEG, FEI, Hillsboro, OR, USA). The TG data were obtained by TG-209 F3 TG analyzer (NETZSCH-Gerätebau GmbH, Selb, Germany). Quantum yields were calculated by using quinine sulfate (Φ_fl_) 0.55 in 0.5 M H_2_SO_4_ solution as a standard. Fluorescence lifetime spectrum was obtained using time-correlated single-photon counting method.

### 2.2. Synthesis

#### 2.2.1. Synthesis of Intermediate **CPBI_0_**

According to the literature [33,34,35], 20 mL polyphosphoric acid (PPA) and 3,3′-diaminobenzidine **1** were added into a 50 mL flask and heated at 160 °C for 2 h. After they were completely dissolved, 3.0 mmol *trans*-butenedioic acid (*E*-BA) **2** was added, and the mixture was heated to 170 °C for 48 h. After cooling to room temperature, the pH was adjusted to alkaline with NaOH solution, and then a blue–black solid was obtained by the filtration.

#### 2.2.2. Synthesis of Series of **CPBI_n_**

According to reported methods [36,37,38], 1 mmol **CPBI_0_**, different molar *n*-C_5_H_11_Br (Table 1), 10 mL MeCN, and moderate NaOH were added into the reaction flask. After refluxing for 24 h, the organic solvent was evaporated in vacuo. The crude product was washed several times with plenty of water to remove NaOH, and then the alkylation product was treated with a mixture of methylene chloride and ethanol. Once the organic phase was collected continuously, the solvent was removed in vacuo. After the desired product was dried in a vacuum drying oven at 40 °C for 24 h, the purified soluble alkylation solid product **CPBI*_n_*** was obtained.

## 3. Results

### 3.1. Structural Characterization and Basic Properties of CPBIs

**Figure 1:**
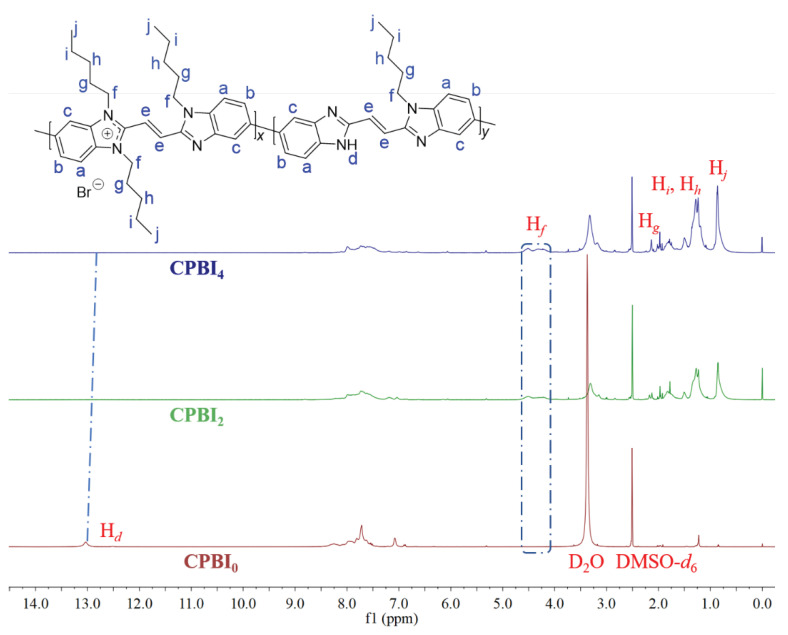
The changes of ^1^H NMR spectra of **CPBI_0_** and **CPBI*_n_***.

The structures of **CPBI_S_** were characterized by ^1^H NMR, IR, XPS, etc. As shown in Figure 1 (see SM for more detailed characterization data of all **CPBIs** from Figures S1–S7), it can be found that, with the *N*-alkylation reaction, the signal of N-H (H*_d_*, 13.03 ppm) in the main chain of **CPBI_0_** is becoming weak for the gradual replacement. At the same time, new signals of alkyl chain characteristics, such as H*_f_* (4.06~4.55 ppm), H*_g_* (1.58~1.65 ppm), H*_i_*, H*_h_* (1.23~1.28 ppm), and H*_j_* (0.686~0.90 ppm), can be observed in **CPBI*_n_*** (Figure S7). In addition, the signal of terminal olefin double bond hydrogen (=CH, 6.32–6.67 ppm) in the polymer was used to calculate the number-average molecular weight (M_n_) of **CPBI*_n_***. As shown in Table S1, it can be seen that, with the increase of the feed ratio of *n*(C_5_H_11_Br)/*n*(**CPBI_0_**), the alkylation rate of **CPBI*_n_*** increases continuously and the M_n_ also increases correspondingly, which is consistent with expectations. Meanwhile, the color of solid product is gradually lightening (Table S1).

In the FT-IR spectra, with the increase of feed ratio [*n*(C_5_H_11_Br)/*n*(**CPBI_0_**)], the benzimidazole N-H stretching vibration at 3300 cm^−1^ is gradually decreased. On the contrary, some characteristic stretching vibrations of saturated alkyl chains at 2984, 2853, 1479, and 730 cm^−1^ are strengthened continuously. Of course, the C–N stretching vibration at 1325 cm^−1^ is also gradually enhanced (Figure S8). These all indicate that the *N*-alkylation reaction has successfully attached the alkyl chain to the polymer via C–N bond.

As reference method [39,40,41], the XPS energy spectra of **CPBI_2_** shown in Figure S9 were analyzed. It can be seen from Figure S9a that 284.76, 399.76, and 531.76 eV are attributed to C1s, N1s, and O1s on the **CPBI_2_** skeleton, respectively. The sub-peaks of C1s are 284.865 eV, 284.346 eV, 285.982 eV, and 287.795 eV, respectively, belonging to C–C/C=C, C–N/C–O, C=N, and the polymer terminal C=O (Figure S9b). The electron energies of C–N and C=N can also be obtained from the sub-peaks of N1s (Figure S9c). Thus, the XPS data further demonstrate the construction of the polymer skeleton. In a word, **CPBI_0_** has been successfully *N*-alkylated, which is proved by ^1^H NMR, IR, and XPS. Additionally, changing the feed ratio [*n*(C_5_H_11_Br)/*n*(**CPBI_0_**)], **CPBI*_n_*** polymers with different alkylation degrees can be obtained, as shown in Table S1. Subsequently, SEM, TG, and XRD of **CPBI*_n_*** were studied and discussed.

The crystallization performance of serial **CPBIs** was analyzed by XRD (Figure S10). Under low alkylation rate (**CPBI_1_**~**CPBI_4_**), the polymer is amorphous. However, with the continuous increase of alkylation rate (**CPBI_5_**~**CPBI_7_**), the polymer morphology tends to become regular, and the crystallinity is increased (Figure S10b). The thermal stabilities of the **CPBIs** were investigated under an O_2_ atmosphere (Figure S11). The initial and termination temperatures of thermal decomposition of **CPBI_5_**~**CPBI_7_** polymers with high alkylation rate (Table S1) and certain crystallinity are significantly higher than those of amorphous **CPBI_1_**~**CPBI_4_** polymers with low alkylation rate (Table S2, and the detailed data analysis can be seen in SM).

**Figure 2:**
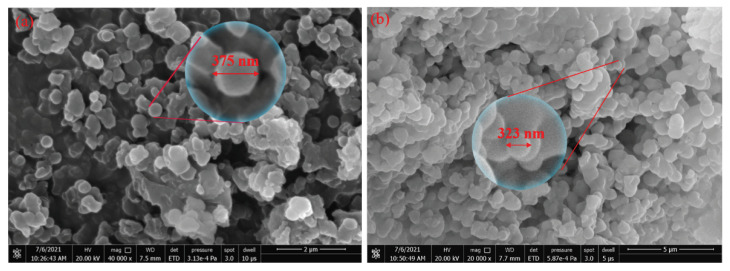
SEM imgaes of **CPBI_2_** (**a**) and **CPBI_4_** (**b**).

Using reported method [42,43,44], SEM was used to observe the morphology of series **CPBIs**, as shown in Figure S12. The surface of **CPBI_0_** is flat and lamellar. When the feed ratio [*n*(C_5_H_11_Br)/*n*(**CPBI_0_**)] is 1/1, the original lamellar structure is destroyed and gradually transferred to the nano-microsphere structure. When the feed ratio is 2/1, **CPBI_2_** has a nano-microsphere structure with a diameter of 375 nm (Figure 2a). With the continuous increase of alkylation rate, there is still nanosphere structure, e.g., CPBI_4_ with a diameter of 323 nm (Figure 2b). When *n*(C_5_H_11_Br)/*n*(**CPBI_0_**) is equal to or bigger than 5:1, the nanosphere becomes a larger microsphere (e.g., **CPBI_5_** with a diameter of 7 μm), and begins to be destroyed and accumulated (see Figure S12 in SM). This may be due to the electrostatic repulsion of the polymers being different with the increase of the alkylation rate during the *N*-alkylation process of **CPBI_0_** and the size of microspheres formed by these polymers under the influence of different electrostatic forces. Therefore, not only can the alkylation rate of **CPBI_0_** be regulated by the feed ratio of reactants, but also, the morphology of the alkylation product can be adjusted, and the nanosphere structure may be formed when the ratio is appropriate (Figure 2).

### 3.2. The Photophysical Properties of Serial CPBIs

**Figure 3:**
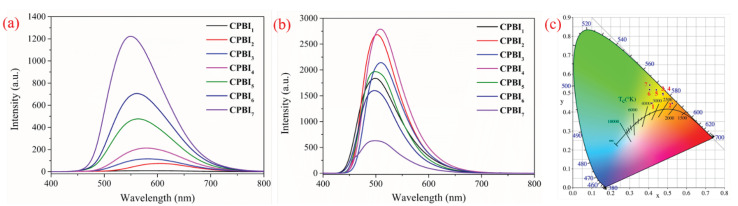
Solid fluorescence emission spectra (**a**), liquid fluorescence emission spectra (1 mg/15 mL in DMSO, λ_ex_ = 375 nm) (**b**), and solid chromaticity diagram of **CPBI*_n_*** with different feeding ratios (**c**).

As an electron-donating group, the alkyl chain easily produces a p–π conjugate with the benzene ring, resulting in enhanced fluorescence of the probe [31]. In addition, for conjugated polymers, the Aggregation-Caused Quenching (ACQ) effect is weakened by the introduction of long alkyl chains. This improves the solid-state fluorescence of the polymer. In order to illustrate the effect of alkylation rate on the fluorescence intensity of the polymer, the fluorescence test of **CPBI*_n_*** in solid and liquid was carried out, respectively, as shown in Figure 3. It can be found that the fluorescence of solid-state **CPBI*_n_*** is greatly influenced by the alkylation rate, and its fluorescence emission intensity increases with the increase of the alkylation rate (Figure 3a). With a significant blue shift, the solid fluorescence color changes from orange–yellow to yellow (Figure 3c and Table S3).

Usually, the use of large conjugated polybenzimidazole in the probe field is limited because of solubility. Here, we modified **CPBI_0_** by *N*-alkylation to obtain a series of new polymer **CPBI*_n_***. These polymers (especially **CPBI_1_**~**CPBI_4_**) are soluble in a variety of organic solvents, such as THF, DMF, DMSO, etc. Among them, DMSO has the best solubility, so DMSO is used as the solvent in the subsequent fluorescence tests. In Figure 3b, it can be found that the fluorescence intensity of **CPBI*_n_*** solution is not only affected by the electron donation of alkyl chain but also greatly related to its structure, solubility, and molecular vibration [32,45,46]. Therefore, there is no obvious regular change in the fluorescence intensity of their solution. Even so, among them, **CPBI_2_** and **CPBI_4_** have relatively strong fluorescence, which may be determined by their nanosphere structure (Figure 2).

The effects of different solvents such as THF, MeCN, EtOH, DMF, DMSO, CH_2_Cl_2,_ and CHCl_3_ on the fluorescence properties of the polymer were also investigated. The experimental results are shown in Figure S13. It can be seen that, in different solvents, the difference in fluorescence intensity of **CPBI_1_**~**CPBI_4_** is mainly affected by solubility rather than solvent polarity. Additionally, the position of the fluorescence emission peak of **CPBI*_n_*** has little relation with the polarity of the solvent.

The metal ions are soluble in water, and they are generally tested in the experiments as salt solutions. In order to eliminate the influence of the presence of water on the detection of metal ions, the changes in the fluorescence intensity of **CPBI_1_**~**CPBI_4_** with different water content were studied (Figure S14). With the increase of water content as DMSO/H_2_O (*v*/*v*), the fluorescence emission intensity of **CPBI*_n_*** is decreased sharply and basically reaches complete quenching (ACQ) at 50% water content (50:50, *v*/*v*). Therefore, to avoid the interference of higher water content, DMSO/H_2_O (90:10, *v*/*v*) is used as the mixed solvent in the following experiments to study the sensing performance of the polymer towards metal ions.

### 3.3. Sensing Performance of CPBI_n_ towards Metal Ions

**Figure 4:**
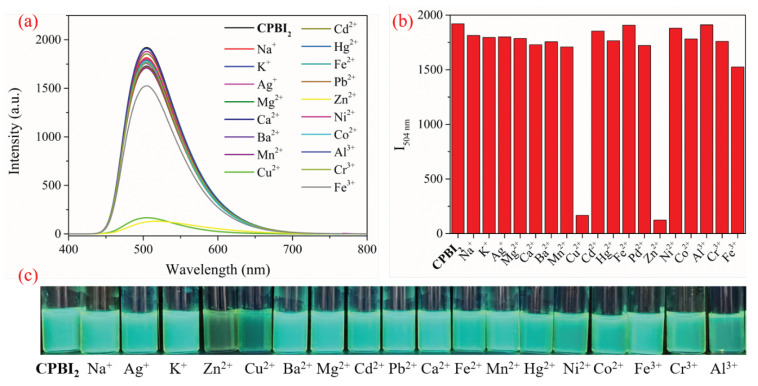
The fluorescence selectivity study of different metal ions (100 μM) in **CPBI_2_** (1 mg/15 mL in DMSO/H_2_O, *v*/*v*, 9/1) solution, λ_ex_ = 375 nm (**a**); the influence of adding different metal ions (100 μM) on **CPBI_2_** fluorescence intensity at 504 nm (**b**); and the fluorescence color change of the solution when different metal ions are added under a 365 nm UV lamp (**c**).

The solubility and photophysical properties of **CPBI_0_** are improved by *N*-alkylation reaction. In DMSO/H_2_O (90:10, *v*/*v*) solvents, the effect of alkylation rate on the fluorescence properties of the polymer was analyzed by studying the corresponding **CPBI*_n_*** to different metal ions. The selectivity of **CPBI_2_** (37.4% alkylation) for 18 metal ions was investigated in DMSO/H_2_O system. Only Cu^2+^ and Zn^2+^ are tightly bound by probe **CPBI_2_**, resulting in the fluorescence quenching of **CPBI_2_** at λ_em_ = 504 nm with the excitation at 375 nm (Figure 4a), and the fluorescence quenching rate is 89.4% and 91.5%, respectively. Compared with the response of Cu^2+^, after the addition of Zn^2+^, the fluorescence color of **CPBI_2_** probe solution is changed from bright green to yellow under 365 nm UV light (Figure 4c), and λ_em_ is red-shifted from 504 nm to 519 nm. For the other metals evaluated, only Fe^3+^ has a slightly similar effect, but this can be negligible in comparison with Cu^2+^ and Zn^2+^ (Figure 4b). Therefore, **CPBI_2_** can be used as a “turn-off” probe for the simultaneous detection of Cu^2+^ and Zn^2+^.

**Figure 5:**
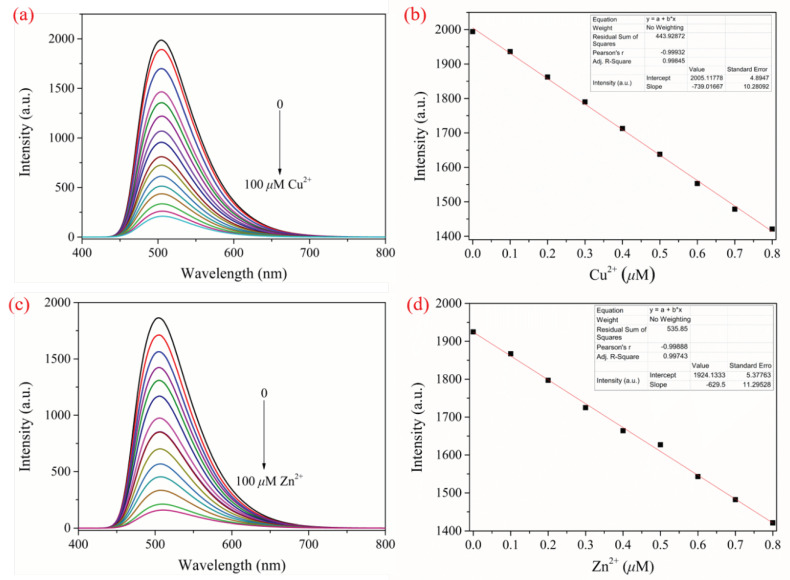
Fluorescence titration of **CPBI_2_** (1 mg/15 mL in DMSO/H_2_O, *v*/*v*, 9/1) solution with Cu^2+^ (**a**,**b**), Zn^2+^ (**c**,**d**), and their corresponding detection limits (λ_ex_ = 375 nm).

We also studied the possibility of other common metal ions interfering with the detection of Cu^2+^ and Zn^2+^. As seen in Figure S15, the presence of other metal ions has no effect on the detection of Cu^2+^ and Zn^2+^ by the probe **CPBI_2_**. The results show that **CPBI_2_** has good anti-interference ability for the detection of Cu^2+^ and Zn^2+^. The fluorescence intensity of **CPBI_2_** is gradually decreased at λ_em_ = 504 nm with the addition of Cu^2+^ and Zn^2+^ (0~100 μM), as shown in Figure 5a,c. According to the calculation method reported in the literature [47,48,49], the fluorescence detection limit of sensor **CPBI_2_** for Cu^2+^ and Zn^2+^ can be calculated as 5.98 × 10^−9^ M and 6.02 × 10^−9^ M, respectively (Figure 5b,d). Compared with the data reported before, the results indicate that sensor **CPBI_2_** is more sensitive than most known Cu^2+^ and Zn^2+^ sensors [50,51,52,53,54] (more comparisons can be seen in Tables S5 and S6).

**Figure 6:**
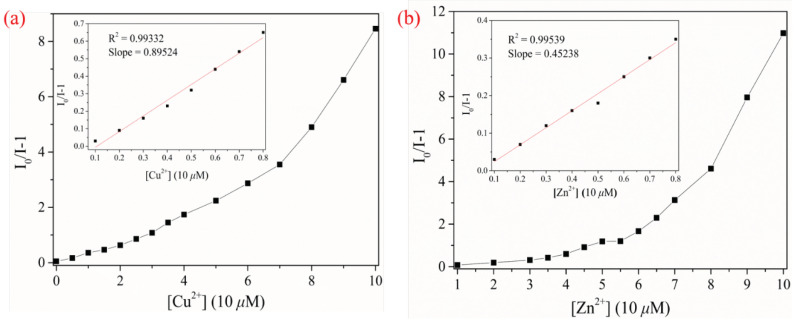
Stern–Volmer diagram of the interaction of sensors **CPBI_2_**, with Cu^2+^ (**a**) and Zn^2+^ (**b**) (the illustration shows the Stern–Volmer linear diagram at low concentrations).

In addition, as a “turn-off” fluorescence sensor, the fluorescence-quenching process of **CPBI_2_** can be determined by Stern–Volmer (S–V) constant (K*sv*), whose calculation formula is: I_0_/I = 1+K*sv*[Q] (Figure 6). The S–V curve has a tendency to bend upward when a higher concentration of Cu^2+^ or Zn^2+^ is added to **CPBI_2_** solution. However, the Stern–Volmer equation has a linear-fitting relationship when the concentration of metal ions is lower. This indicates that there are static and dynamic quenching processes of **CPBI_2_** interaction with metal ions. K*sv* values for **CPBI_2_** towards Cu^2+^ and Zn^2+^ can be calculated to be 8.95 × 10^4^ and 4.52 × 10^4^ M^−1^, respectively. Fluorescence sensors with dynamic quenching may show good reversibility detection for metal ions [55,56].

Similarly, we also conducted the fluorescence tests on other alkylated polymers, such as **CPBI_1_**, **CPBI_3,_** and **CPBI_4_**, as shown in Figures S16–S28. As anticipated, **CPBI_1_**, **CPBI_3,_** and **CPBI_4_** can selectively recognize Cu^2+^ and Zn^2+^ (Figures S16, S20, and S24). For example, it can be found that the Stern–Volmer equation of **CPBI_4_** has a good linear relationship with the concentration of metal ions (K*sv* values for **CPBI_4_** towards Cu^2+^ and Zn^2+^ can be calculated to be 2.20 × 10^4^ and 1.45 × 10^4^ M^−1^, respectively), so the fluorescence quenching mode of **CPBI_4_** is static quenching (Figure S28).

Importantly, the sensitivity to metal ions is increasing with the increase of alkylation rate (Table S4). However, the fluorescence-quenching effect is decreasing. This may be due to the increase in electron-donating groups in the polymer as the alkylation rate increases, which makes it easier to combine with electron-deficient metal ions. Nevertheless, the steric hindrance of the polymer is increased likewise, and the combination of metal ions is reduced correspondingly when the alkylation rate is too high, so the fluorescence-quenching effect is reduced. This represents that the different alkylation rates will lead to the different detection performances of polymer fluorescent probes, which further demonstrates the regulation of alkyl chain on the performance of polymer fluorescent probes.

### 3.4. Sensing Mechanism of CPBIs for Metal Ions

**Figure 7:**
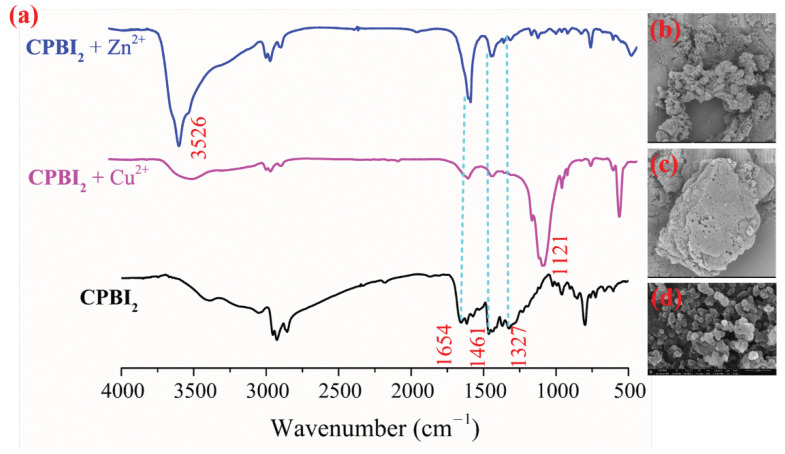
FT-IR spectra (**a**), SEM images of **CPBI_2_** before and after the addition of Cu^2+^ or Zn^2+^ ((**b**): **CPBI_2_**+Zn^2+^; (**c**): **CPBI_2_**+Cu^2+^; (**d**): **CPBI_2_**).

According to the reported method [53,57,58,59], we explored the sensing mechanism of **CPBIs** before and after the interaction with analytes by IR, SEM, and DFT. Using **CPBI_2_** as an example, in the IR spectra (Figure 7a), the C=N stretching vibration (1654 cm^−1^), C–N stretching vibration (1327 cm^−1^), and N-H stretching vibration of imidazole ring in probe **CPBI_2_** are greatly weakened after the addition of Cu^2+^. This suggests that Cu^2+^ can interact with C=N and not alkylated C–N in the imidazole ring of the **CPBI_2_** backbone. Of course, after complexing with metal ions, there is an aggregation for **CPBI_2_**, which limits the movement of the alkyl chain, resulting in an obvious weakening of the saturated C-H flexural vibration (1461 cm^−1^) also. Importantly, a new absorption can be observed at 1121 cm^−1^ when **CPBI_2_** interacts with Cu^2+^. It is consistent with the results in the literature [16,60,61,62]. In addition, the results of SEM further demonstrate that the original nanoparticle structure of **CPBI_2_** (Figure 7d) has aggregated into a massive flaky structure (Figure 7c) after the interaction of **CPBI_2_** with Cu^2+^.

Similarly, the C–N stretching vibration (1327 cm^−1^) of **CPBI_2_** is also greatly weakened when **CPBI_2_** interacts with Zn^2+^, while the C=N stretching vibration intensity (1654 cm^−1^) is basically unchanged (Figure 7a). This shows that Zn^2+^ is mainly coordinated with C–N in the main chain of benzimidazole. Additionally, the flexural vibration of alkyl chain (1461 cm^−1^) is also limited when coordinated with Zn^2+^. Moreover, a new absorption appears at 3540 cm^−1^, which is also consistent with the results in the literature [63,64]. At the same time, the image of SEM (Figure 7b) also shows the interaction between **CPBI_2_** and Zn^2+^. Therefore, the interaction between **CPBI_2_** and Cu^2+^ and Zn^2+^ can be proved by both IR and SEM. For other series of **CPBI*_n_*** polymers, such as **CPBI_1_**, **CPBI_3,_** and **CPBI_4_**, their IR and SEM morphologies after the interaction with metal ions are similar (Figures S29–S31 in SM).

In addition, we further investigated the quenching process of the probe by changing the fluorescence lifetime [65]. It can be found that the presence of metal ions has no effect on the decay of the fluorescence lifetime of **CPBI_2_** and **CPBI_4_** when the concentration of metal ions is low (Figure S32 in SM). Therefore, it is also verified that the fluorescence-quenching process of **CPBI_2_** and **CPBI_4_** is static quenching in the low concentration range of metal ions.

**Figure 8:**
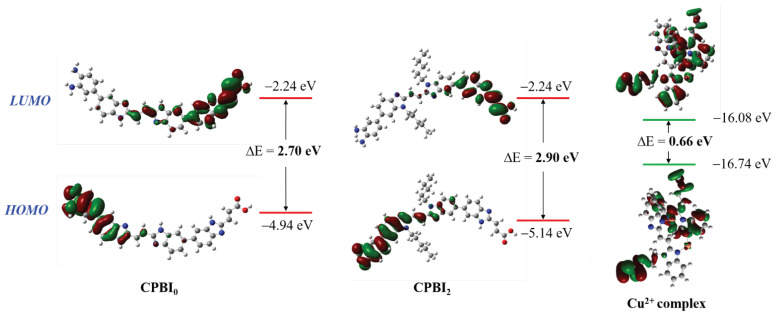
The optimized geometries of **CPBI_2_**, its Cu^2+^ complex, and **CPBI_0_** (B3LYP/6-31G basis set **CPBI_0_**, **CPBI_2_**, and B3LYP/6-31G basis set for C, H, N; LanL2DZ for Cu^2+^ in complex).

Using the reported method [66,67,68,69], Gauss DFT-B3LYP/6-31G method was used to optimize the structure of **CPBI_2_**, **CPBI_2_**–Cu^2+^ complex, and **CPBI_0_**. As shown in Figure 8, the energy of the lowest unoccupied molecular orbital (LUMO) before and after alkylation (**CPBI_0_**) is mainly distributed on the carboxylic acid group side at the end of the polymer, and the energy is −2.24 eV. On the contrary, the energy of the highest occupied molecular orbital (HOMO) is mainly distributed on the side containing NH_2_ at the end of the polymer. After the introduction of the alkyl chain, only HOMO is slightly affected, which makes the HOMO-LUMO band gap (∆E) slightly larger. The structure of the complex combined with metal ions was optimized by the Cu^2+^ complex. It can be found that the ∆E is greatly reduced when **CPBI_2_** is complexed with Cu^2+^ (Figure 8). This indicates that a stable metal complex has been formed, which is further proof of coordination between metal ions and polymers [70,71].

### 3.5. Logic Gate Construction

**Figure 9:**
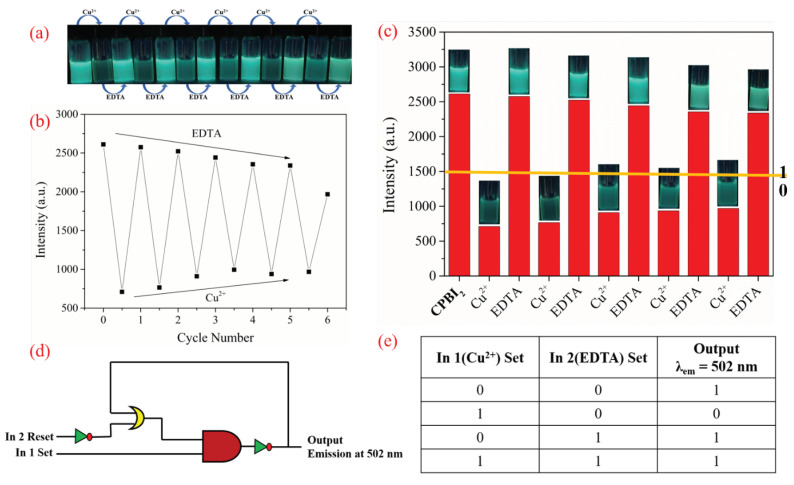
Fluorescence experiment of the probe **CPBI_2_** in cyclic use: alternately adding Cu^2+^ and EDTA into the **CPBI_2_** solution (λ_ex_ = 375 nm), the color change diagram of the solution under the 365 nm UV lamp (**a**); the fluorescence intensity cycle fluorescence diagram (**b**); input (In 1 = Cu^2+^ and In 2 = EDTA) histogram (**c**); implication logic gate (**d**); corresponding truth table (**e**).

The reversibility of the sensor is an important indicator of whether the sensor can be reused multiple times. **CPBI_2_** was selected for the logic gate cycle experiment (Figure 9) because of its good fluorescence-quenching effect and obvious detection phenomenon when sensing copper ions. According to the fluorescence titration experiment, when EDTA is added to **CPBI_2_**–Cu^2+^ complex, Cu^2+^ in the complex will bind to EDTA due to the stronger complexing ability of EDTA so that **CPBI_2_** becomes free and the corresponding fluorescence intensity is also recovered (Figure 9b). In addition, this fluorescent “off/on” switching behavior can be observed at 365 nm UV lamp (Figure 9a). As a chemical sensor, **CPBI_2_** has shown good stability and reversibility; it is expected to be designed as a chemical sensor based on molecular logic gate [72,73].

Due to the above fluorescence quenching–restoration cycle of probe **CPBI_2_** with Cu^2+^ and EDTA, a logic gate can be implemented on Boolean logic operations (Figure 9d). This is an IMPLICATION logic gate displaying memory unit with two inputs (In 1 and In 2) and one output (Figure 9e). We set Cu^2+^ and EDTA to inputs In 1 and In 2, respectively. Their presence and absence are denoted as 1 and 0. The output signal is the emission intensity at 504 nm, and the threshold is set to 1500 a.u. (Figure 9c). When the fluorescence intensity is higher than the threshold, the output signal is on (1). Additionally, when the fluorescence intensity is below the threshold, the output signal is off (0). Based on the above basic logic gate, when both Cu^2+^ and EDTA are present or absent, the output signal is on (1). When In 1 and In 2 are in the state (1, 0), the output signal is off (0). When In 1 and In 2 are in the state (0, 1), the output signal is on (1) (Figure 9e). Therefore, the IMPLICATION logic gate can be constructed at the molecular level by monitoring the emission intensity value at 504 nm of the output signal through the input signal (Cu^2+^ and EDTA) [16,74,75,76].

### 3.6. Visual Detection of Cu^2+^ in Solid State

**Figure 10:**
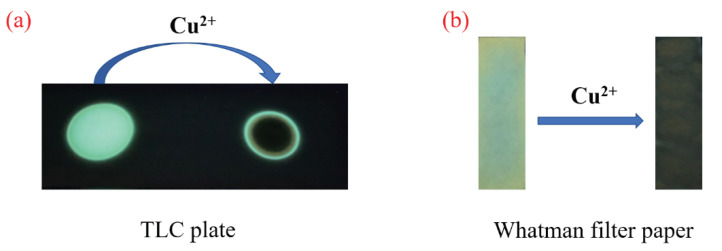
Fluorescence image (under 365 nm UV light) of sensor **CPBI_2_** adsorbed on a TLC plate with a spot of Cu^2+^ solution on sensor **CPBI_2_** (**a**); fluorescence changes (under 365 nm UV lamp) of test strips for detecting Cu^2+^ in aqueous solution (**b**).

According to the literature method [50,77,78], the thin layer chromatography (TLC) plate with **CPBI_2_** solution adsorbed shows green fluorescence under 365 nm UV lamp. The fluorescence in the center of **CPBI_2_** visible to the naked eye is rapidly quenched when Cu^2+^ solution is added to the TLC plate adsorbed with **CPBI_2_**, as shown in Figure 10a. Therefore, the TLC plate adsorbed with **CPBI_2_** can be used to detect Cu^2+^, and its visual detection of Cu^2+^ in solution can be achieved.

In addition, according to the method reported in the literature [47,79], the Whatman test paper is immersed in DMSO solution containing **CPBI_2_**, and then vacuum drying makes **CPBI_2_** test paper. The test paper shows green fluorescence under the 356 nm UV lamp, and the fluorescence is quickly quenched when Cu^2+^ solution is added to the test paper (Figure 10b). Therefore, **CPBI_2_** can be made into not only solution-coated thin layer chromatography plates but also Whatman test paper to realize solid-state visual detection of Cu^2+^.

## 4. Conclusions

In summary, a novel large conjugation polybenzimidazole compound **CPBI_0_** was synthesized by a simple green-metal-free catalytic reaction. In order to improve its solubility and fluorescence properties, a series of different alkylated polymers **CPBI*_n_*** were obtained by *N*-alkylation reaction. An alkyl chain is an electron-donor group; the difference in the alkylation rate means that the number of electron donors in the polymer is different, resulting in a different binding ability with electron-deficient metal ions. Therefore, although **CPBI*_n_***-series polymers (**CPBI_1_**~**CPBI_4_**) show a good recognition ability for Cu^2+^ and Zn^2+^, they have different sensitivities, indicating the regulation of the alkyl chain on the performance of a polymer fluorescent probe. In addition, **CPBI_2_** with a nano-microsphere can be designed as an ultra-sensitive IMPLICATION logic gate at the molecular level to cyclically detect Cu^2+^. Furthermore, TLC plates and test papers containing **CPBI_2_** have been developed for the solid visual detection of Cu^2+^ in a solution. This study provides a new idea and method for simple synthesis and fluorescence regulation of multifunctional fluorescent probes.

## Figures and Tables

**Table 1:** The effects of different feed ratios on the basic structure of serial **CPBI*_n_***.

Sample	Feed Ratio (RBr/CPBI_0_)	R^1^	R^2^	R^3^	R^4^	R^5^
**CPBI_1_**	1:1	*n*-C_5_H_11_-	H	*n*-C_5_H_11_-	H	-
**CPBI_2_**	2:1	*n*-C_5_H_11_-	*n*-C_5_H_11_-	*n*-C_5_H_11_-	H	-
**CPBI_3_**	3:1	*n*-C_5_H_11_-	*n*-C_5_H_11_-	*n*-C_5_H_11_-	H	-
**CPBI_4_**	4:1	*n*-C_5_H_11_-	*n*-C_5_H_11_-	*n*-C_5_H_11_-	*n*-C_5_H_11_-	*n*-C_5_H_11_-
**CPBI_5_**	5:1	*n*-C_5_H_11_-	*n*-C_5_H_11_-	*n*-C_5_H_11_-	*n*-C_5_H_11_-	*n*-C_5_H_11_-
**CPBI_6_**	6:1	*n*-C_5_H_11_-	*n*-C_5_H_11_-	*n*-C_5_H_11_-	*n*-C_5_H_11_-	*n*-C_5_H_11_-
**CPBI_7_**	7:1	*n*-C_5_H_11_-	*n*-C_5_H_11_-	*n*-C_5_H_11_-	*n*-C_5_H_11_-	*n*-C_5_H_11_-

## Data Availability

Data can be made available from the authors upon reasonable request.

## Supplementary Materials

The following are available online at https://www.mdpi.com/article/10.3390/polym13183091/s1, Figure S1. ^1^H NMR spectrum of CPBI_0_; Figure S2. ^1^H NMR spectrum of CPBI_1_; Figure S3. ^1^H NMR spectrum of CPBI_2_; Figure S4. ^1^H NMR spectrum of CPBI_3_; Figure S5. ^1^H NMR spectrum of CPBI_4_; Figure S6. ^1^H NMR spectrum of CPBI_5_; Figure S7. The changes of ^1^H NMR spectra of CPBIs with different molar feed ratios [*n*(C_5_H_11_Br)/*n*(CPBI_0_)]; Figure S8. The FT-IR spectra of CPBIs with different molar feed ratios [*n*(C_5_H_11_Br)/*n*(CPBI_0_)]; Figure S9. The full XPS spectra of CPBI_2_ (a) and its C1s (b), N1s (c) peaks; Figure S10. The XRD analysis of CPBIn with different molar feed ratios [*n*(C_5_H_11_Br)/*n*(CPBI_0_)]; Figure S11. TG analysis of CPBIs with different molar feed ratios [*n*(C_5_H_11_Br)/*n*(CPBI_0_)]; Figure S12. SEM analysis of CPBI*_n_* with different molar feed ratios; Figure S13. Fluorescence emission spectra in different polar solvents (*λex* = 375 nm) of CPBI_1_ (a), CPBI_2_ (b), CPBI_3_ (c) and CPBI_4_ (d); Figure S14. Fluorescence emission spectra in DMSO solutions (1 mg/15 mL) with different water content as DMSO/H_2_O (*v*/*v*) of CPBI_1_ (a), CPBI_2_ (b), CPBI_3_ (c) and CPBI_4_ (d), *λex* = 375 nm; Figure S15. Fluorescence emission spectra of sensor CPBI_2_ solution (1 mg/15 mL in DMSO/H_2_O, *v*/*v*, 9/1) with Zn^2+^ (100 μM) and other metal ions (100 μM) (a) and comparison of fluorescence quenching rate (b), fluorescence emission spectra of Cu^2+^ (100 μM) and other metal ions (c) and comparison of fluorescence quenching rate (d), *λex* = 375 nm; Figure S16. The fluorescence selectivity study of different metal ions (100 μM) in CPBI_1_ (1 mg/15 mL in DMSO/H_2_O, *v*/*v*, 9/1) solution (a), the influence of adding different metal ions (100 μM) on CPBI_1_ fluorescence intensity at 500 nm (b), *λex* = 375 nm; Figure S17. Fluorescence emission spectra of sensor CPBI1 solution (1 mg/15 mL in DMSO/H_2_O, *v*/*v*, 9/1) with Cu^2+^ (100 μM) and other metal ions (100 μM) (the upper), fluorescence emission spectra of Zn^2+^ (100 μM) and other metal ions (100 μM) (the lower), λex = 375 nm; Figure S18. Fluorescence emission spectra of CPBI1 (1 mg/15 mL in DMSO/H_2_O, *v*/*v*, 9/1) solution with different concentrations of Cu^2+^ and the linear relationship between CPBI_1_ and low concentrations of Cu^2+^, λex = 375 nm; Figure S19. Fluorescence emission spectra of CPBI_1_ (1 mg/15 mL in DMSO/H_2_O, *v*/*v*, 9/1) solution with different concentrations of Zn^2+^ and the linear relationship between CPBI_1_ and low concentrations of Zn^2+^, λex = 375 nm; Figure S20. The fluorescence selectivity study of different metal ions (100 μM) in CPBI_3_ (1 mg/15 mL in DMSO/H_2_O, *v*/*v*, 9/1) solution (a), the influence of adding different metal ions (100 μM) on CPBI_3_ fluorescence intensity at 510 nm (b), λex = 375 nm; Figure S21. Fluorescence emission spectra of sensor CPBI_3_ solution (1 mg/15 mL in DMSO/H_2_O, *v*/*v*, 9/1) with Cu^2+^ (100 μM) and other metal ions (100 μM) (the upper), fluorescence emission spectra of Zn^2+^ (100 μM) and other metal ions (100 μM) (the lower), λex = 375 nm; Figure S22. Fluorescence emission spectra of CPBI_3_ (1 mg/15 mL in DMSO/H_2_O, *v*/*v*, 9/1) solution with different concentrations of Cu^2+^ and the linear relationship between CPBI_3_ and low concentrations of Cu^2+^, λex = 375 nm; Figure S23. Fluorescence emission spectra of CPBI_3_ (1 mg/15 mL in DMSO/H_2_O, *v*/*v*, 9/1) solution with different concentrations of Zn^2+^ and the linear relationship between CPBI_3_ and low concentrations of Zn^2+^, λex = 375 nm; Figure S24. The fluorescence selectivity study of different metal ions (100 μM) in CPBI4 (1 mg/15 mL in DMSO/H_2_O, *v*/*v*, 9/1) solution, λex = 375 nm (a), the influence of adding different metal ions (100 μM) on CPBI4 fluorescence intensity at 510 nm (b) and the color change of the solution (c); Figure S25. Fluorescence emission spectra of sensor CPBI_4_ solution (1 mg/15 mL in DMSO/H_2_O, *v*/*v*, 9/1) with Zn^2+^ (100 μM) and other metal ions (100 μM) (a) and comparison of fluorescence quenching rate (b), fluorescence emission spectra of Cu^2+^ (100 μM) and other metal ions (100 μM) (c) and comparison of fluorescence quenching rate (d), λex = 375 nm; Figure S26. Fluorescence emission spectra of CPBI4 (1 mg/15 mL in DMSO/H_2_O, *v*/*v*, 9/1) solution with different concentrations of Cu^2+^ and the linear relationship between CPBI4 and low concentrations of Cu^2+^, λex = 375 nm; Figure S27. Fluorescence emission spectra of CPBI_4_ (1 mg/15 mL in DMSO/H_2_O, *v*/*v*, 9/1) solution with different concentrations of Zn^2+^ and the linear relationship between CPBI_4_ and low concentrations of Zn^2+^, λex = 375 nm; Figure S28. Stern-Volmer diagram of the interaction of sensors CPBI4 with Cu^2+^ (a) and Zn^2+^ (b) (the illustration shows the Stern-Volmer linear diagram at low concentrations); Figure S29. FT-IR spectra (a), SEM images of CPBI_1_ before and after the addition of Cu^2+^ or Zn^2+^ (b: CPBI_1_ + Zn^2+^; c: CPBI_1_ + Cu^2+^; d: CPBI_1_); Figure S30. FT-IR spectra (a), SEM images of CPBI_3_ before and after the addition of Cu2+ or Zn^2+^ (b: CPBI_3_ + Zn^2+^; c: CPBI_3_ + Cu^2+^; d: CPBI_3_); Figure S31. FT-IR spectra (a), SEM images of CPBI_4_ before and after the addition of Cu^2+^ or Zn^2+^ (b: CPBI_4_ + Zn^2+^; c: CPBI_4_+ Cu^2+^; d: CPBI_4_); +; Figure S32. Time-correlated single photon counting (TCSPC) plot for CPBI_2_ (left) and CPBI_4_ (right) interacted with Cu^2+^ and Zn^2+^ (λex = 375 nm, λem = 504 nm for CPBI_2_ and λem = 510 nm for CPBI_4_, respectively). Table S1. The effects of different feed ratios on yield, color and actual alkylation rate of CPBIn; Table S2. The thermal decomposition temperatures of CPBIs with different feed ratios; Table S3. Photophysical properties of CPBIn with different feed ratios. Table S4. Comparison of LOD when the sensor CPBI*_n_* detects Cu^2+^ and Zn^2+^; Table S5. The comparison of probe CPBIn with the reported Cu^2+^ probes in solution; Table S6. The comparison of probe CPBI*_n_* with the reported Zn^2+^ probes in solution.
