# Supplementary material for: Preparation of Large Conjugated Polybenzimidazole Fluorescent Materials and Their Application in Metal Ion Detection
Source: Polymers (Basel). 2021 Sep 14;13(18):3091. doi: 10.3390/polym13183091 (PMC8472194; doi:10.3390/polym13183091)
Supplement: Supplementary file 1 [file polymers-13-03091-s001.zip › polymers-1365537-supplementary.pdf]

Supplementary Materials

Preparation of Large Conjugated Polybenzimidazole Fluorescent Materials and their Application in Metal Ion Detection

Xi-Ying Cao[1], Chu-Ming Pang[1,2,*], Ying Xiao[1], Wan-Qing Xiao[1], Shi-He Luo[1,*], Jin-Ping He[1], Zhao-Yang Wang[1,*]

[1] *School of Chemistry, South China Normal University, Key Laboratory of Theoretical Chemistry of Environment, Ministry of Education; Guangzhou Key Laboratory of Analytical Chemistry for Biomedicine, Guangzhou 510006, P. R. China.*

[2] *School of Health Medicine, Guangzhou Huashang College, Guangzhou 511300, P. R. China.*

* Corresponding Author: Zhao-Yang Wang, 19951088@m.scnu.edu.cn. Tel: 8620-39310258, luoshihescnu@m.scnu.edu.cn, 2017021874@m.scnu.edu.cn.

Contents

1. Experimental section........3
2. Characterization spectra and data of **CPBIs** (**Figures. S1-S6**)........5
3. The ^{1}H NMR spectra of **CPBIs** with different molar feed ratios (**Figure S7**)........11
4. The FT-IR spectra of **CPBIs** with different molar feed ratios (**Figure S8**)........12
5. The full XPS spectra of **CPBI$_2$** and its C1s, N1s peaks (**Figure S9**)........13
6. The effects of different feed ratios on serial **CPBI$_n$** (**Table S1**)........14
7. The XRD analysis of **CPBIs** with different molar feed ratios (**Figure S10**)........15
8. TG analysis of serial **CPBIs** with different molar feed ratios (**Figure S11** and **Table S2**)........16
9. The SEM analysis of **CPBIs** with different molar feed ratios (**Figure S12**)........18
10. Fluorescence spectra of **CPBI$_n$** with different alkylation ratios (**Table S3**)........19
11. Effects of different solvent systems on the fluorescence properties of **CPBI$_n$** (**Figure S13**)........20
12. Effects of different water content on the fluorescence properties of **CPBI$_n$** (**Figure S14**)........21
13. The competitive experiments of **CPBI$_2$** for Cu^{2+} or Zn^{2+} (**Figure S15**)........22
14. The selective experiments of **CPBI$_1$** towards metal ions (**Figure S16**)........23
15. The competitive experiments of **CPBI$_1$** for Cu^{2+} or Zn^{2+} (**Figure S17**)........24

16. Fluorescent titration and detection limit of **CPBI$_1$** for Cu^{2+} (**Figure S18**)......................................25
17. Fluorescent titration and detection limit of **CPBI$_1$** for Zn^{2+} (**Figure S19**).......................................26
18. The selective experiments of **CPBI$_3$** towards metal ions (**Figure S20**)..27
19. The competitive experiments of **CPBI$_3$** for Cu^{2+} or Zn^{2+} (**Figure S21**)..28
20. Fluorescent titration and detection limit of **CPBI$_3$** for Cu^{2+} (**Figure. S22**).....................................29
21. Fluorescent titration and detection limit of **CPBI$_3$** for Zn^{2+} (**Figure S23**).......................................30
22. The selective experiments of **CPBI$_4$** towards metal ions (**Figure S24**)..31
23. The competitive experiments of **CPBI$_4$** for Cu^{2+} or Zn^{2+} (**Figure S25**)..32
24. Fluorescent titration and detection limit of **CPBI$_4$** for Cu^{2+} (**Figure S26**)......................................33
25. Fluorescent titration and detection limit of **CPBI$_4$** for Zn^{2+} (**Figure S27**).......................................34
26. The plots of **CPBI$_4$** *vs* concentration of Cu^{2+} and Zn^{2+} (**Figure S28**)...35
27. Comparison of detection limits of Cu^{2+} and Zn^{2+} by serial **CPBI$_n$** (**Table S4**)..............................36
28. The changes of FT-IR spectra and morphology before or after the combination with analytes (**Figures S29-S31**)..37
29. TCSPC plots for **CPBI$_2$** and **CPBI$_4$** interacted with Cu^{2+} and Zn^{2+} (**Figure S32)**…..........................39
30. Comparison of **CPBI$_n$** with Cu^{2+} and Zn^{2+} probes available in the literature (**Table S5** and **Table S6)**...40

1. Experimental section

1.1. Chemicals and apparatus

All reagents and organic solvents were purchased from commercial suppliers and used without further purification. ^1H NMR spectra of compounds were recorded on a Bruker 600 MHz instrument. Fluorescence spectra were carried out by a Hitachi F-4600 fluorescence spectrometer. The Scanning Electron Microscope was obtained by FEI Quanta 250 FEG field emission scanning electron microscope. The TG data were obtained by TG-209 F3 TG analyzer. Quantum yields were calculated by using quinine sulfate (Φ_{fl}) 0.55 in 0.5 M H_2SO_4 solution as a standard. Fluorescence lifetime spectrum was obtained using time correlated single photon counting method.

1.2. Synthesis

1.2.1. Synthesis of intermediate CPBI$_0$

According to the method in the reference [1-3], 20 mL polyphosphoric acid (PPA) and 3,3'-di-aminobenzidine **1** were added into a 50 mL flask and heated at 160 ^oC for 2 h. After they were completely dissolved, 3.0 mmol *trans*-butenedioic acid (*E*-BA) **2** was added and the mixture was heated to 170 ^oC for 48 h. After cooling to room temperature, the pH was adjusted to alkaline with NaOH solution, and then a blue-black solid was obtained by the filtration.

1.2.2. Synthesis of series CPBI$_n$

According to the method of reference [4-6], 1 mmol **CPBI$_0$**, different molar *n*-$C_5H_{11}Br$, 10 mL MeCN and moderate NaOH were added into the reaction flask. After refluxing for 24 hours, the organic solvent was evaporated *in vacuo*. The crude product was washed several times with plenty of water to remove NaOH, and then the alkylation product was treated by the mixture of methylene chloride and

ethanol. Once the organic phase was collected continuously, and the solvent was removed *in vacuo*. After the desired product was dried in a vacuum drying oven at 40 °C for 24 h, the purified soluble alkylation solid product **CPBI*n*** was obtained.

1.3. Methods

1.3.1. General procedure for optical spectral measurements

The **CPBI*n*** samples were dissolved in DMSO to acquire stock solution. Then, **CPBI*n*** test solution (DMSO/H_2O, *V*/*V* = 9/1) was prepared for fluorescence emission spectra at room temperature. Among them, 1 mg sample was dissolved in 15 mL mixed solvent.

1.3.2. Limit of detection

As the reported method [7], the limit of detection (LOD) was measured by the equation: LOD = $3\delta/K$. Therein, δ is the standard deviation of the blank measurements (n = 10), and the K is the slope of the calibration curve.

1.3.3. SEM analysis

According to the reported method [8-10], the morphology for **CPBIs** and the morphology changes of **CPBI*n*** combined with metal ions were determined by field emission scanning electron microscope.

1.3.4. XPS analysis

As the reported method [11-13], the combing energy of N1s, O1s and C1s in the product were measured by Axis Ultra-DLD X-ray photoelectron spectrometer.

2. Characterization spectra and data of $CPBI_0$ and serial $CPBI_n$

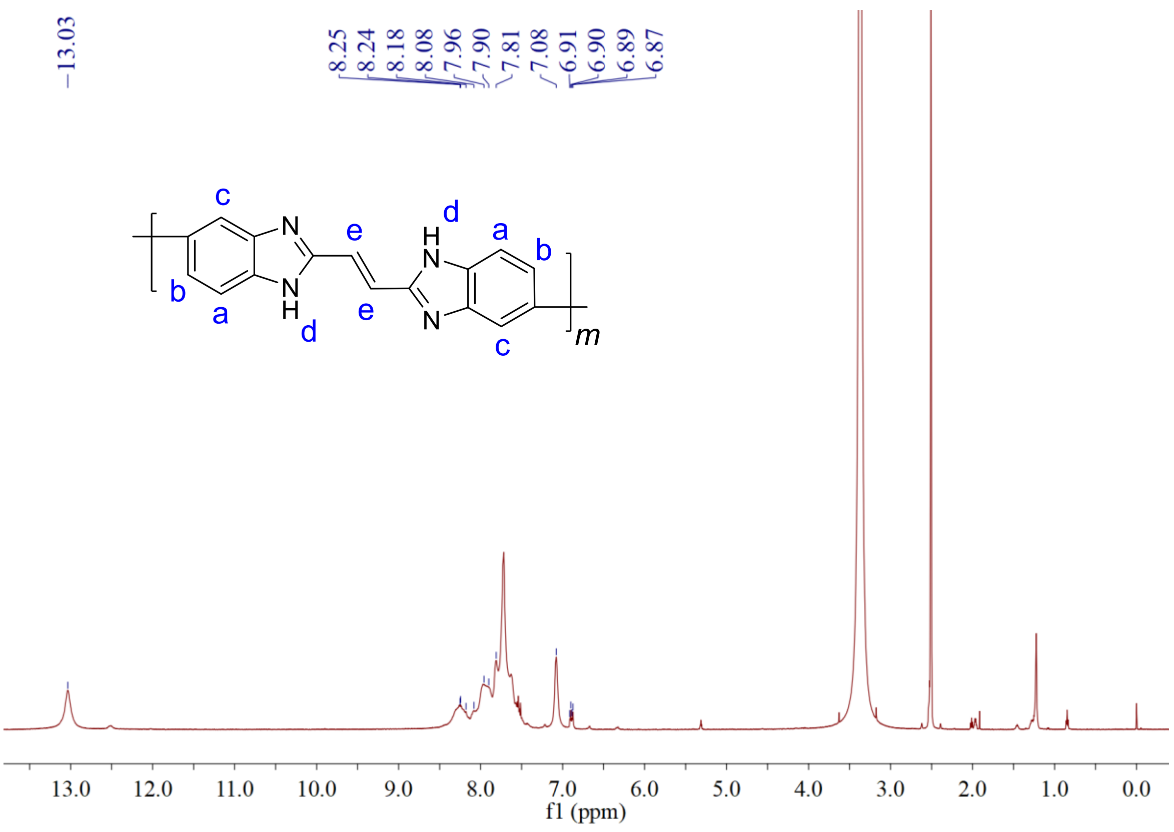

Figure S1. ^{1}H NMR spectrum of **$CPBI_0$**.

$CPBI_0$, blue-black solid, yield 82.1%; ^{1}H NMR (600 MHz, DMSO-d_6, δ, ppm): 6.87-7.08 (*m*, H_e, =CH in main chain), 7.81-7.90 (*m*, H_a, Ar-H in benzimidazole unit of main chain), 7.96-8.08 (*m*, H_b, Ar-H in benzimidazole unit of main chain), 8.18-8.25 (*m*, H_c, Ar-H in benzimidazole unit of main chain), 13.03 (*s*, H_d, NH in benzimidazole unit of main chain); IR (KBr), ν, cm^{-1}: 3390, 3059, 1614, 1578, 1534, 1437, 1290, 1209, 956, 855, 802. According to the ^{1}H NMR result, the number-average molecular weight (M_n) of **$CPBI_0$** is calculated to be about 2000 Da.

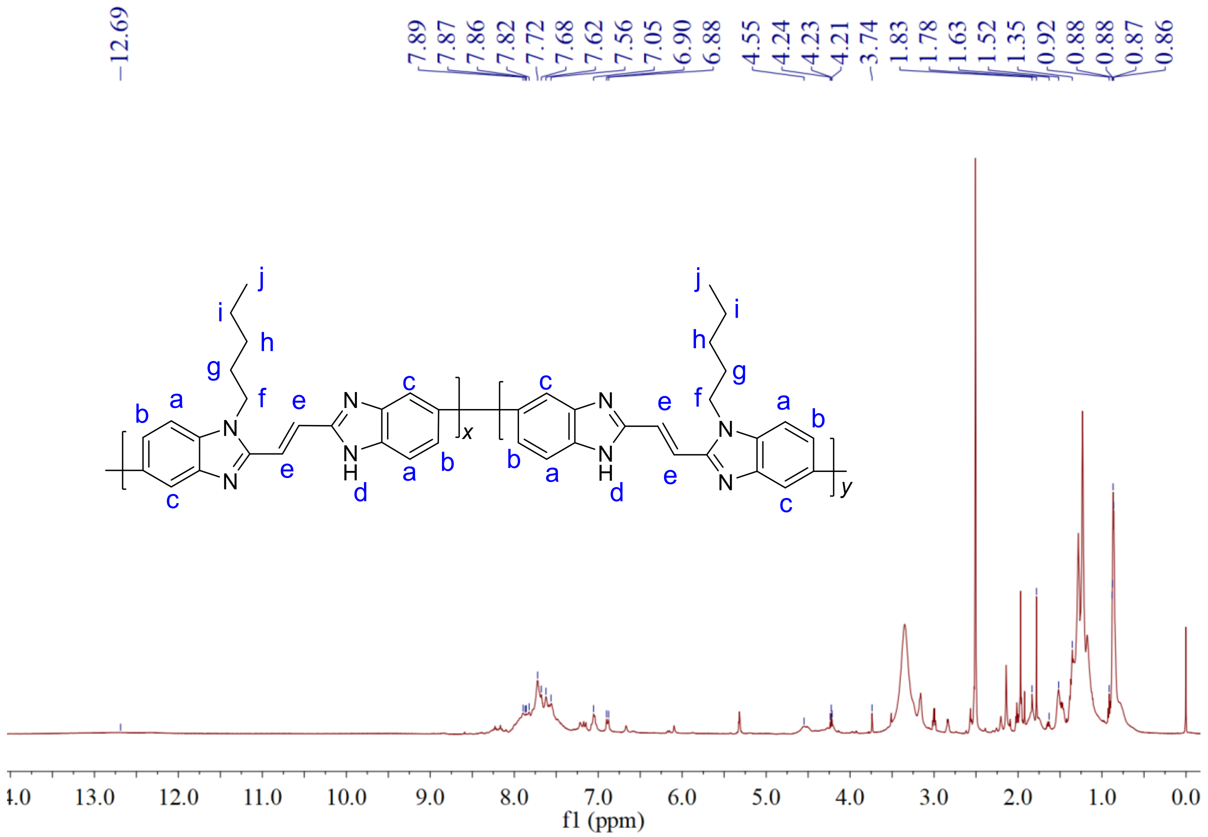

Figure S2. ^{1}H NMR spectrum of **$CPBI_1$**.

$CPBI_1$, brown solid, yield 26.0 %, ^{1}H NMR (600 MHz, DMSO-d_6, δ, ppm): 0.86-0.92 (*m*, H_j, CH_3 in *N*-alkyl chain), 1.35-1.62 (*m*, H_i, H_h, CH_2 in *N*-alkyl chain), 1.78-1.83 (*m*, H_g, CH_2 in *N*-alkyl chain), 3.51-4.55 (*m*, H_f, NCH_2 in *N*-alkyl chain), 6.88-7.21 (*s*, H_e, =CH in main chain), 7.56-7.62 (*m*, H_a, Ar-H in benzimidazole unit of main chain), 7.68-7.72 (*m*, H_b, Ar-H in benzimidazole unit of main chain), 7.82-7.89 (*m*, H_c, Ar-H in benzimidazole unit of main chain), 12.69 (*b*, H_d, NH in benzimidazole unit of main chain). IR (KBr), *v*, cm^{-1}: 3382, 3075, 2954, 2923, 2854, 1659, 1616, 1574, 1466, 1372, 1297, 964, 860, 803. According to the ^{1}H NMR result, the number-average molecular weight (M_n) of **$CPBI_1$** is calculated to be about 2900 Da.

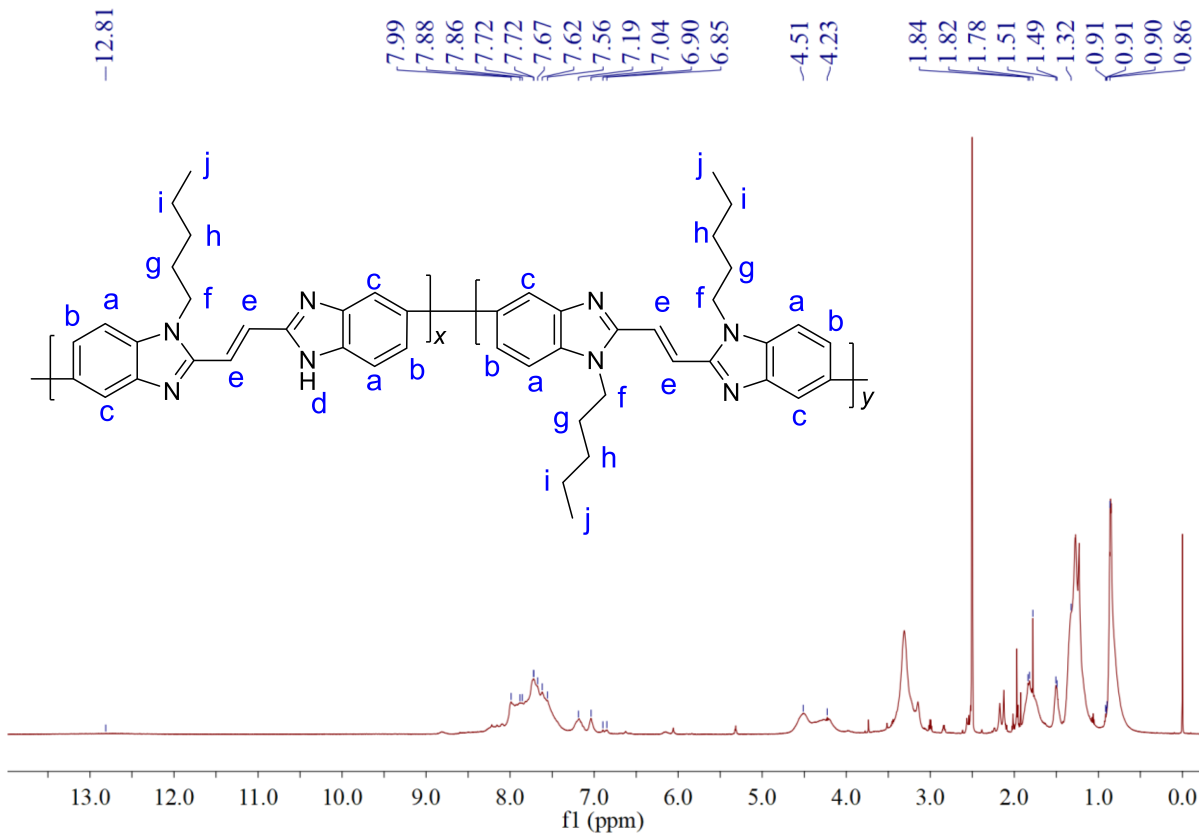

Figure S3. ^{1}H NMR spectrum of **CPBI$_2$**.

CPBI$_2$, brownish-yellow solid, yield 54.2 %, ^{1}H NMR (600 MHz, DMSO-d_6, δ, ppm): 0.86-0.91 (*m*, H_j, CH_3 in *N*-alkyl chain), 1.32-1.51 (*m*, H_h, H_i, CH_2 in *N*-alkyl chain), 1.78-1.84 (*m*, H_g, CH_2 in *N*-alkyl chain), 4.23-4.51 (*m*, H_f, NCH_2 in *N*-alkyl chain), 6.85-7.19 (*s*, H_e, =CH in main chain), 7.56-7.62 (*m*, H_a, Ar-H in benzimidazole unit of main chain), 7.67-7.72 (*m*, H_b, Ar-H in benzimidazole unit of main chain), 7.88-7.99 (*m*, H_c, Ar-H in benzimidazole unit of main chain), 12.81 (*b*, H_d, NH in benzimidazole unit of main chain); IR (KBr), v, cm^{-1}: 3390, 3054, 2955, 2927, 2853, 1654, 1617, 1568, 1461, 1327, 1229, 957, 857, 801, 724. According to the ^{1}H NMR result, the number-average molecular weight (M_n) of **CPBI$_2$** is calculated to be about 3500 Da.

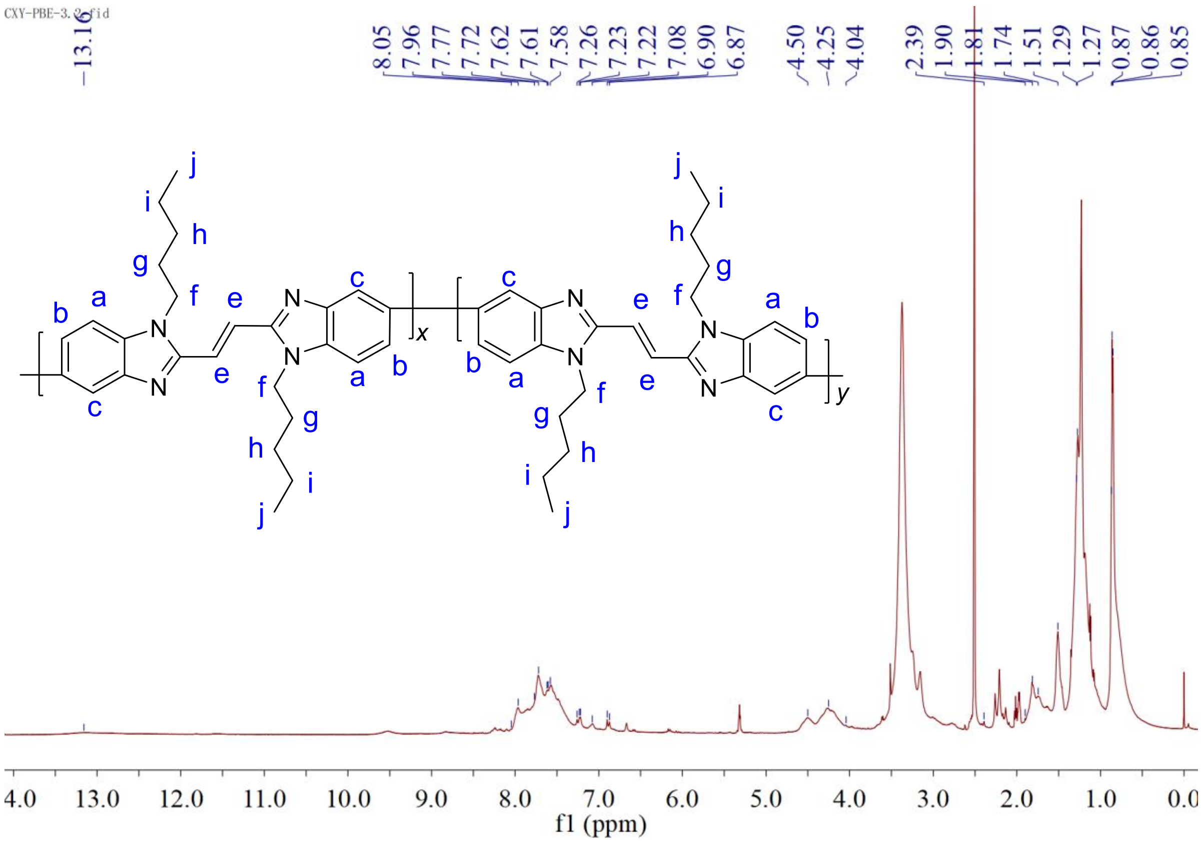

Figure S4. ^{1}H NMR spectrum of **CPBI$_3$**.

CPBI$_3$, orange solid, yield 27.0 %, ^{1}H NMR (600 MHz, DMSO-d_6, δ, ppm): 0.85-0.87 (*m*, H_j, CH_3 in *N*-alkyl chain), 1.27-1.51 (*m*, H_h, H_i, CH_2 in *N*-alkyl chain), 1.74-1.90 (*m*, H_g, CH_2 in *N*-alkyl chain), 4.04-4.50 (*m*, H_f, NCH_2 in *N*-alkyl chain), 6.87-7.26 (*s*, H_e, =CH in main chain), 7.58-7.62 (*m*, H_a, Ar-H in benzimidazole unit of main chain), 7.72-7.77 (*m*, H_b, Ar-H in benzimidazole unit of main chain), 7.96-8.05 (*m*, H_c, Ar-H in benzimidazole unit of main chain), 13.16 (*b*, H_d, NH in benzimidazole unit of main chain); IR (KBr), *v*, cm^{-1}: 3388, 3051, 2957, 2922, 2852, 1663, 1621, 1570, 1467, 1369, 1318, 963, 857, 795, 723. According to the ^{1}H NMR result, the number-average molecular weight (M_n) of **CPBI$_3$** is calculated to be about 3700 Da.

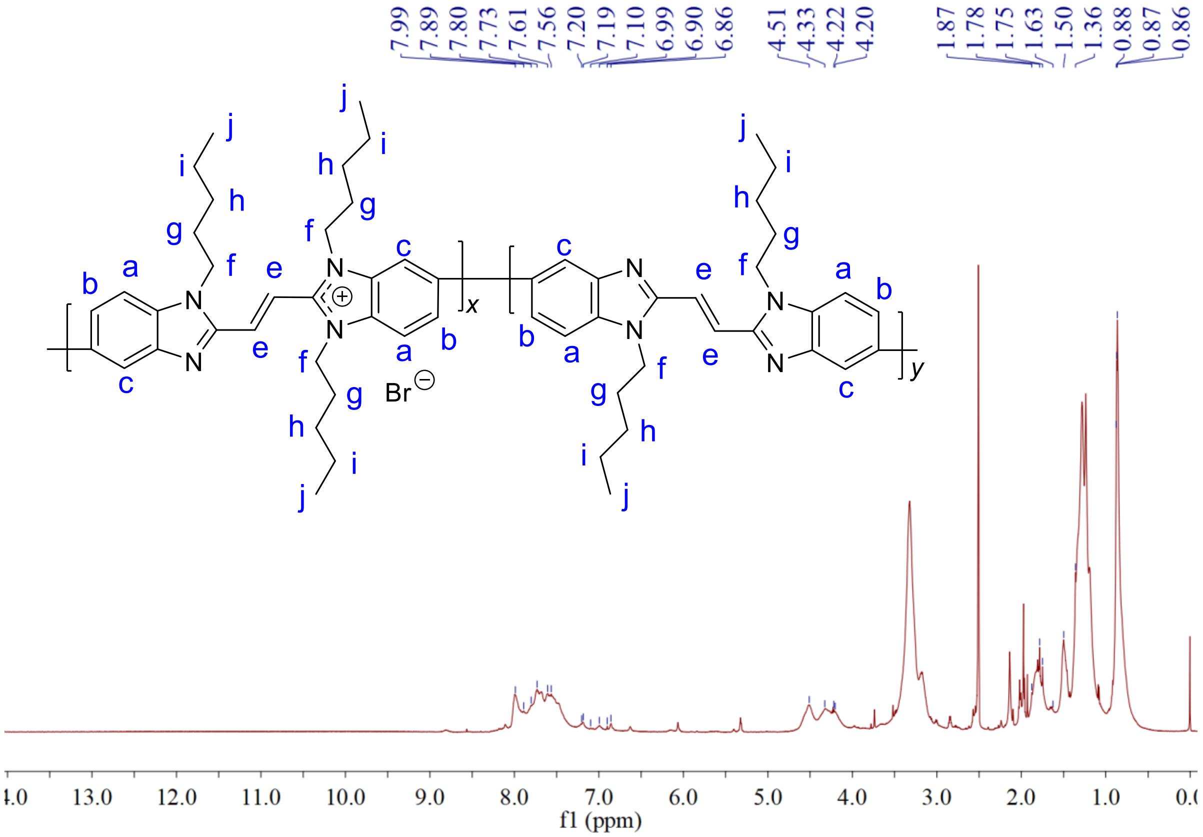

Figure S5. ^{1}H NMR spectrum of **CPBI4**.

CPBI4, orange solid, yield 33.9 %, ^{1}H NMR (600 MHz, DMSO-d_6, δ, ppm): 0.86-0.88 (*m*, H_j, CH_3 in *N*-alkyl chain), 1.36-1.63 (*m*, H_h, H_i, CH_2 in *N*-alkyl chain), 1.75-1.87 (*m*, H_g, CH_2 in *N*-alkyl chain), 4.20-4.51 (*m*, H_f, NCH_2 in *N*-alkyl chain), 6.86-7.20 (*s*, H_e, =CH in main chain), 7.56-7.61 (*m*, H_a, Ar-H in benzimidazole unit of main chain), 7.73-7.80 (*m*, H_b, Ar-H in benzimidazole unit of main chain), 7.89-7.99 (*m*, H_c, Ar-H in benzimidazole unit of main chain); IR (KBr), ν, cm^{-1}: 3375, 3052, 2956, 2925, 2856, 1661, 1615, 1571, 1466, 1371, 1320, 961, 850, 802, 724. According to the ^{1}H NMR result, the number-average molecular weight (M_n) of **CPBI4** is calculated to be about 4300 Da.

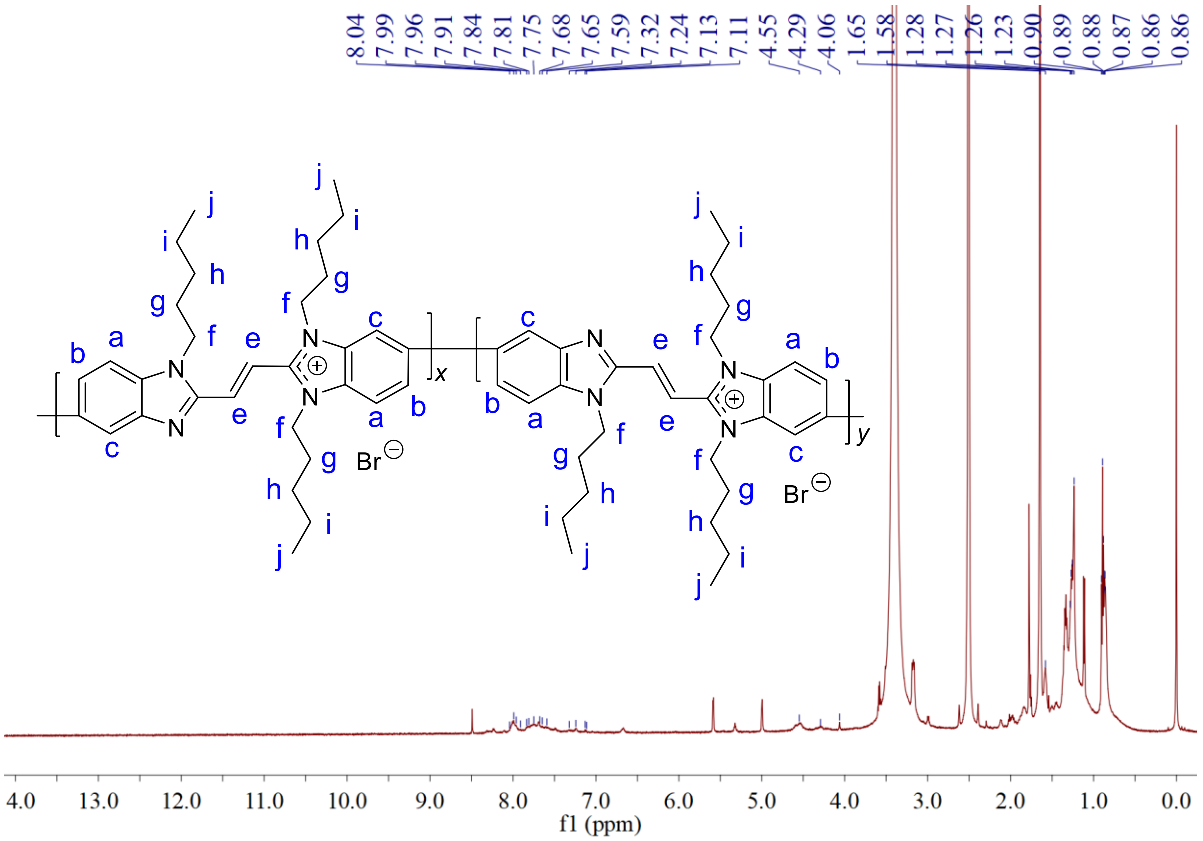

Figure S6. ^{1}H NMR spectrum of **CPBI$_5$**.

CPBI$_5$, yellow solid, yield 77.4 %, ^{1}H NMR (600 MHz, DMSO-d_6, δ, ppm): 0.86-0.90 (*m*, H$_j$, CH$_3$ in *N*-alkyl chain), 1.23-1.28 (*m*, H$_h$, H$_i$, CH$_2$ in *N*-alkyl chain), 1.58-1.65 (*m*, H$_g$, CH$_2$ in *N*-alkyl chain), 4.06-4.55 (*m*, H$_f$, NCH$_2$ in *N*-alkyl chain), 7.11-7.23 (*s*, H$_e$, =CH in main chain), 7.59-7.68 (*m*, H$_a$, Ar-H in benzimidazole unit of main chain), 7.75-7.84 (*m*, H$_b$, Ar-H in benzimidazole unit of main chain), 7.91-8.04 (*m*, H$_c$, Ar-H in benzimidazole unit of main chain), IR (KBr), v, cm^{-1}: 3394, 3003, 2951, 2923, 2850, 1603, 1547, 1450, 1450, 1410, 1324, 1010, 922, 848, 646, 618. According to the ^{1}H NMR result, the number-average molecular weight (M$_n$) of **CPBI$_5$** is calculated to be about 5100 Da.

CPBI$_6$, yellow solid, yield 73.5 %; IR (KBr), v, cm^{-1}: 3424, 3005, 2929, 2862, 2502, 2188 1628, 1587, 1526, 1436, 1392, 1047, 1009, 920, 878, 652, 619.

CPBI$_7$, yellowish solid, yield 74.3 %; IR (KBr), v, cm^{-1}: 3386, 2965, 2937, 2871, 2498, 2188, 1672, 1630, 1541, 1495, 1387, 1046, 1017, 921, 877, 651, 617.

3. The ^{1}H NMR spectra of CPBIs with different molar feed ratios

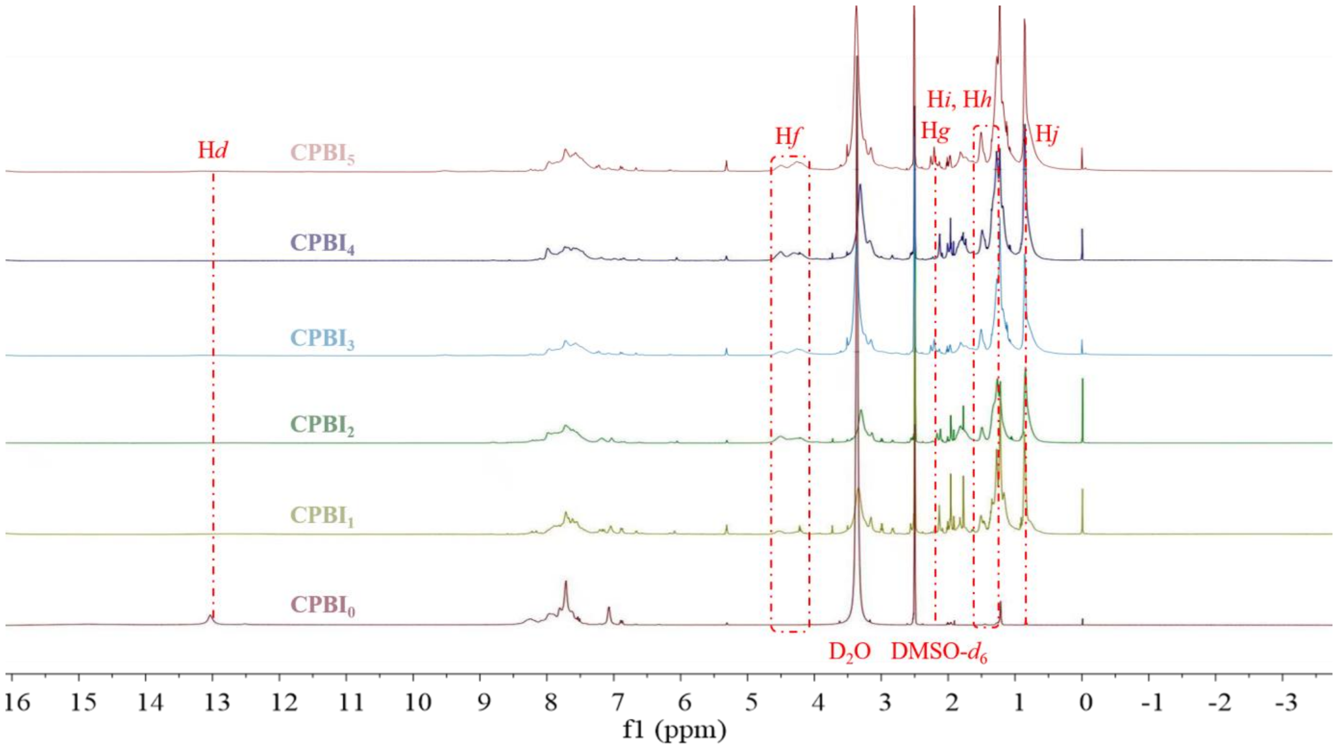

Figure S7. The changes of ^{1}H NMR spectra of **CPBIs** with different molar feed ratios [n($C_5H_{11}Br$)/n(**CPBI$_0$**)].

4. The FT-IR spectra of CPBIs with different molar feed ratios

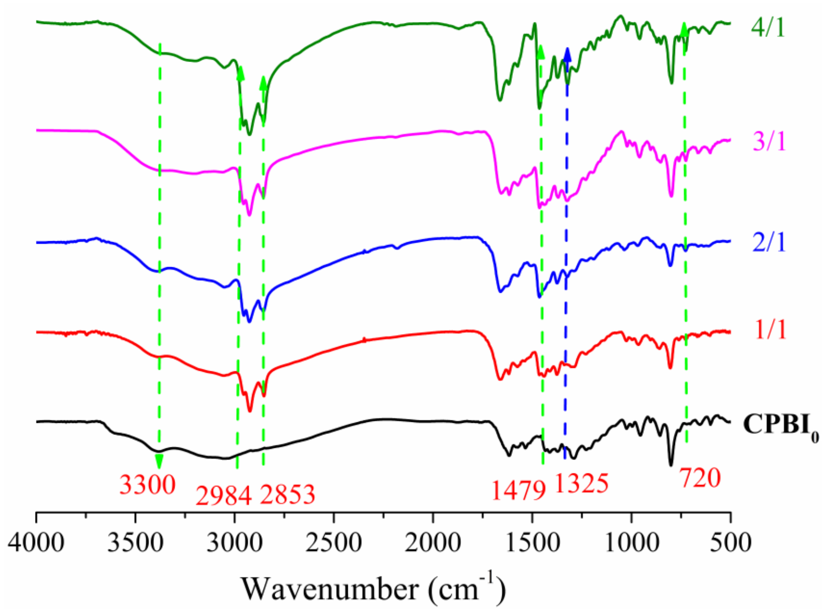

Figure S8. The FT-IR spectra of **CPBIs** with different molar feed ratios [n($C_5H_{11}Br$)/n(**CPBI$_0$**)].

5. The full XPS spectra of $CPBI_2$ and its C1s, N1s peaks

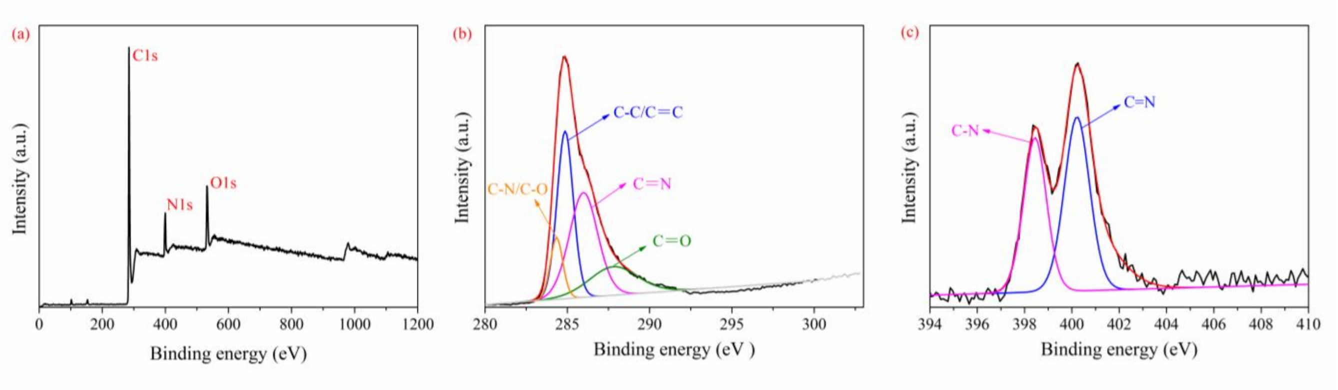

Figure S9. The full XPS spectra of **$CPBI_2$** (a) and its C1s (b), N1s (c) peaks.

6. The effects of different feed ratios on serial CPBI$_n$

Table S1. The effects of different feed ratios on yield, color and actual alkylation rate of **CPBI$_n$**.

Sample No.	Feed ratio (RBr/**CPBI$_0$**)	Yield (%)	Appearance of product	Actual alkylation rate (%)	Mn (Da)
**CPBI$_1$**	1:1	26.0	Brown	23.3	2900
**CPBI$_2$**	2:1	54.2	Brownish yellow	37.4	3500
**CPBI$_3$**	3:1	27.0	Orange yellow	43.7	3700
**CPBI$_4$**	4:1	33.9	Orange yellow	58.2	4300
**CPBI$_5$**	5:1	77.4	Yellow	77.2	5100
**CPBI$_6$**	6:1	73.5	Yellow	-	-
**CPBI$_7$**	7:1	74.3	Yellowish	-	-

7. The XRD analysis of CPBIs with different molar feed ratios

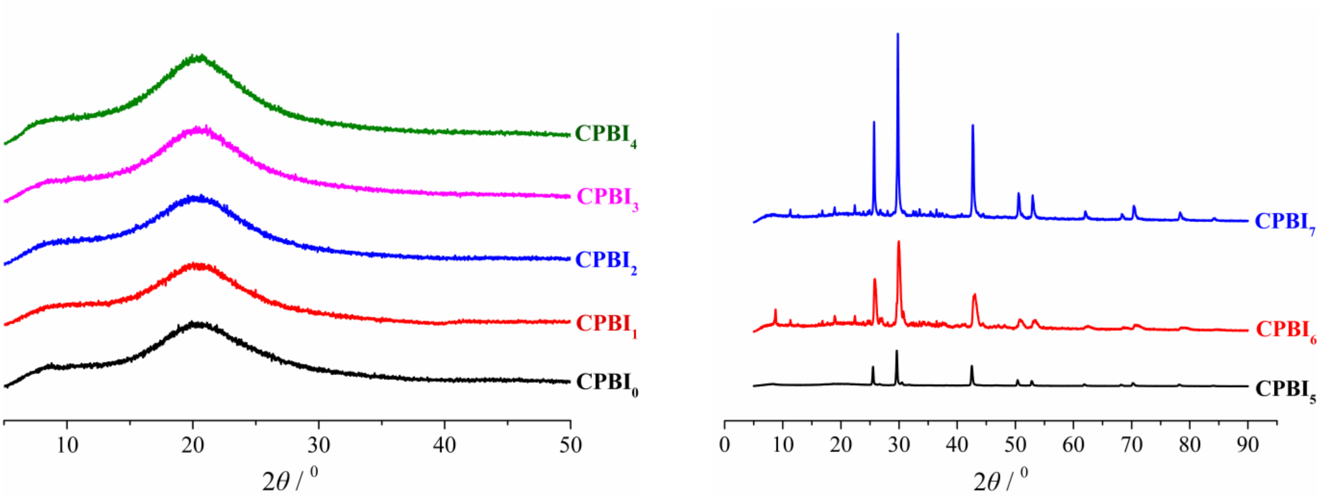

Figure S10. The XRD analysis of **CPBI*n*** with different molar feed ratios [*n*($C_5H_{11}Br$)/*n*(**$CPBI_0$**)].

8. TG analysis of serial CPBIs with different molar feed ratios

The thermal stability of polybenzimidazole modified by alkyl chain needs to be further investigated with the help of thermogravimetric analyzer in O_2 atmosphere. The results are shown as **Figure S11**.

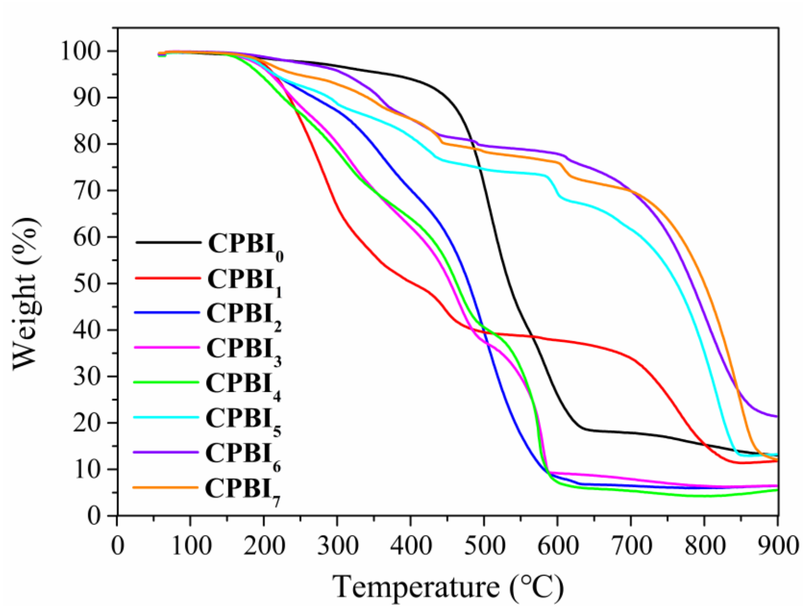

Figure S11. TG analysis of **CPBIs** with different molar feed ratios [$n(C_5H_{11}Br)/n$(**CPBI$_0$**)].

It can be seen that, **CPBI$_0$** itself has high thermal stability, the initial temperature of thermal decomposition is 468.5 °C, the termination temperature is 654.2 °C, and the thermal weight loss rate reaches 90.45% (**Table S2**).

After the *N*-alkylation reaction of **CPBI$_0$**, the initial decomposition temperature of **CPBI$_n$** is significantly reduced. It may be due to that, after the *N*-alkylation reaction, the obtained polymers first decompose the introduced alkyl chain part when the temperature rises, and the decomposition temperature of the alkyl chain is lower than that of the main chain. Therefore, when $n(C_5H_{11}Br)$/ *n*(**CPBI$_0$**) is 1/1, the decomposition temperature is 208.8 °C, and the thermal decomposition temperature continues to decrease with the increase of alkylation rate.

Table S2. The thermal decomposition temperatures of **CPBIs** with different feed ratios.

Feed ratio (RBr/**CPBI$_0$**)	Initial temperature (°C)	Terminal temperature (°C)	Thermo-gravimetric rate (%)
**CPBI$_0$**	468.5	654.2	90.45
1:1	208.8	831.4	87.83
2:1	169.1	574.3	93.28
3:1	164.7	586.7	93.16
4:1	163.2	576.3	93.42
5:1	162.0	838.9	86.00
6:1	173.6	848.2	77.92
7:1	176.5	867.3	87.70

However, once the *N*-alkylation reaction is high enough, the obtained polymer is quaternary ammonium (**Table 1**, in the main text). This enables the polymer to have certain crystallinity (see the corresponding discussion in the main text), leading to the continuous increase of the initial and termination temperature of thermal decomposition of the polymer **CPBI$_5$** ~ **CPBI$_7$**. In particular, the termination temperature of thermal decomposition of polymer increases significantly. For **CPBI$_7$**, the termination temperature of thermal decomposition is as high as 867.3 ^{o}C (**Table S2**). The results are in good agreement with the results of XRD test.

9. The SEM analysis of CPBIs with different molar feed ratios

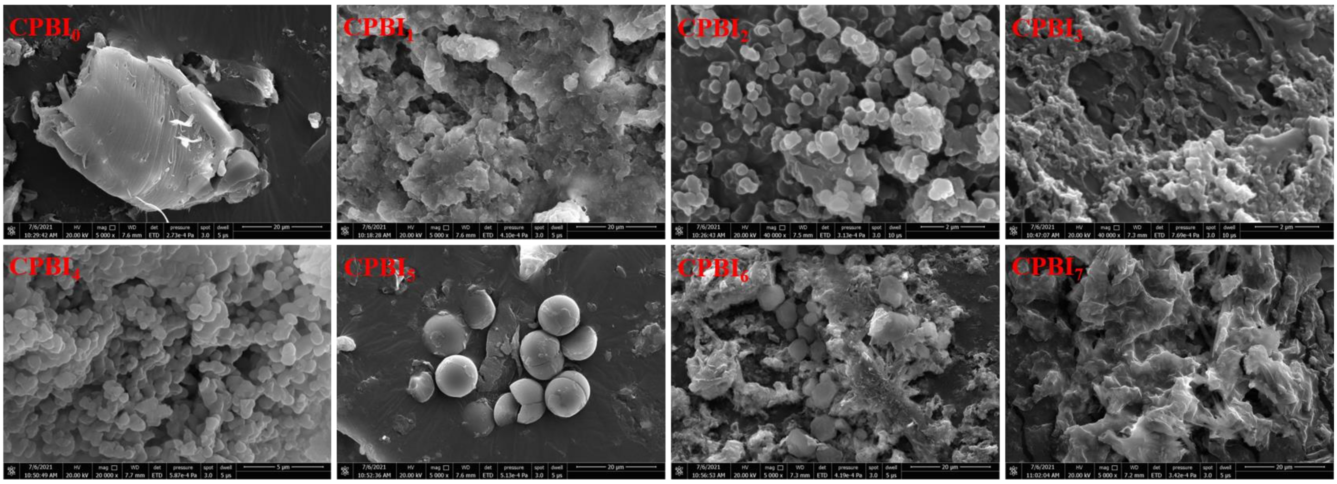

Figure S12. SEM analysis of **CPBI*$_n$*** with different molar feed ratios.

10. Fluorescence spectra of CPBI$_n$ with different alkylation ratios

Table S3. Photophysical properties of **CPBI$_n$** with different feed ratios.

Compounds	Solution				Solid		Photo images
	λ_{abs} [a] (nm)	λ_{em} [b] (nm)	$\Delta\upsilon$ [c] (nm)	Φ_f [d] (%)	λ_{em} [b] (nm)	$\Delta\upsilon$ [c] (nm)	
**CPBI$_1$**	411	498	87	12.43%	582	207	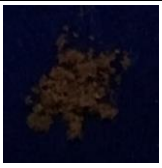
**CPBI$_2$**	413	502	89	16.15%	604	229	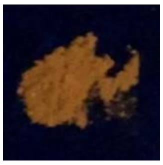
**CPBI$_3$**	412	509	97	13.45%	580	205	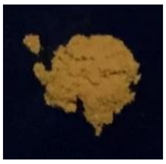
**CPBI$_4$**	410	509	99	21.31%	577	202	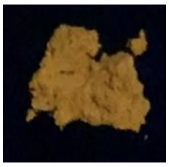
**CPBI$_5$**	411	498	87	13.88%	563	188	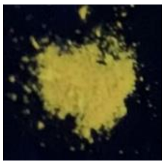
**CPBI$_6$**	414	497	83	11.76%	560	185	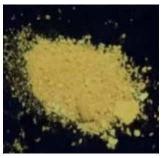
**CPBI$_7$**	417	498	81	8.48%	548	173	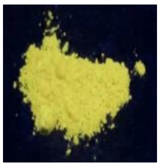

[a] Experimental absorption maxima.

[b] Experimental emission maxima.

[c] Stokes shift.

[d] The fluorescence quantum yields.

11. Effects of different solvent systems on the fluorescence properties of CPBI*n*

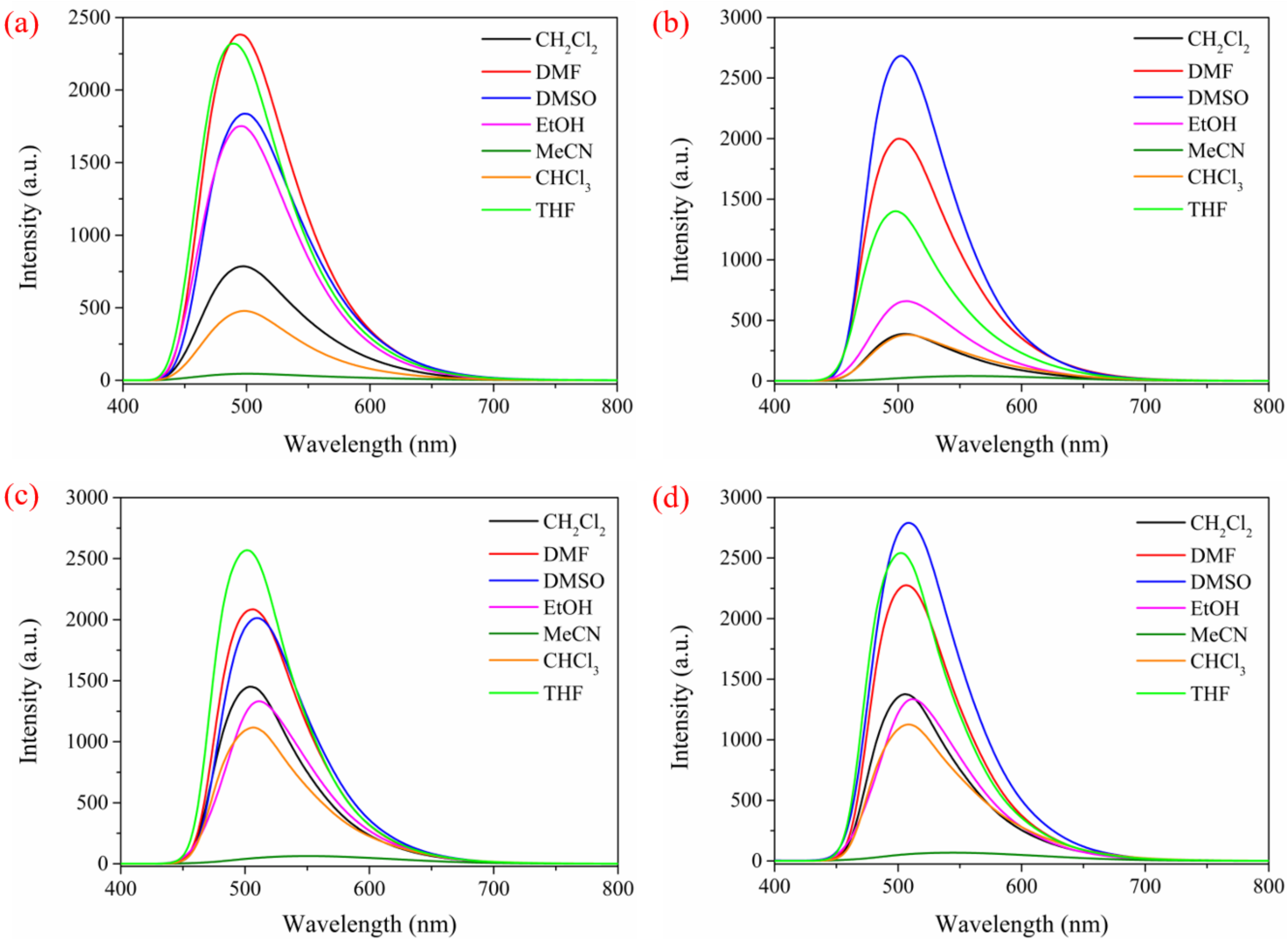

Figure S13. Fluorescence emission spectra in different polar solvents (λ_{ex} = 375 nm) of **CPBI1** (a), **CPBI2** (b), **CPBI3** (c) and **CPBI4** (d).

12. Effects of different water content on the fluorescence properties of CPBI*n*

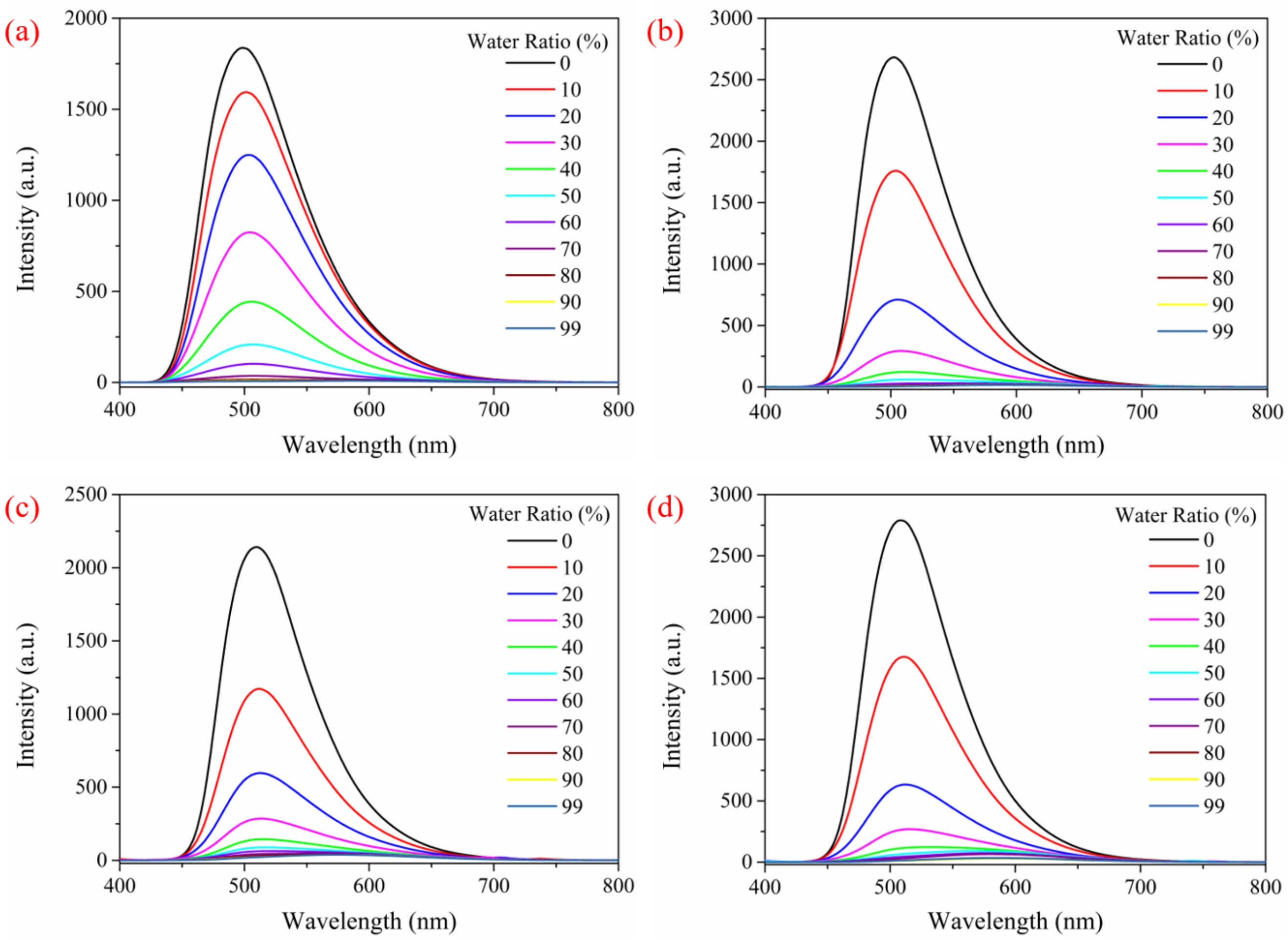

Figure S14. Fluorescence emission spectra in DMSO solutions (1 mg/15 mL) with different water content as DMSO/H_2O (v/v) of **CPBI$_1$** (a), **CPBI$_2$** (b), **CPBI$_3$** (c) and **CPBI$_4$** (d), λ_{ex} = 375 nm.

13. The competitive experiments of CPBI₂ for Cu^{2+} or Zn^{2+}

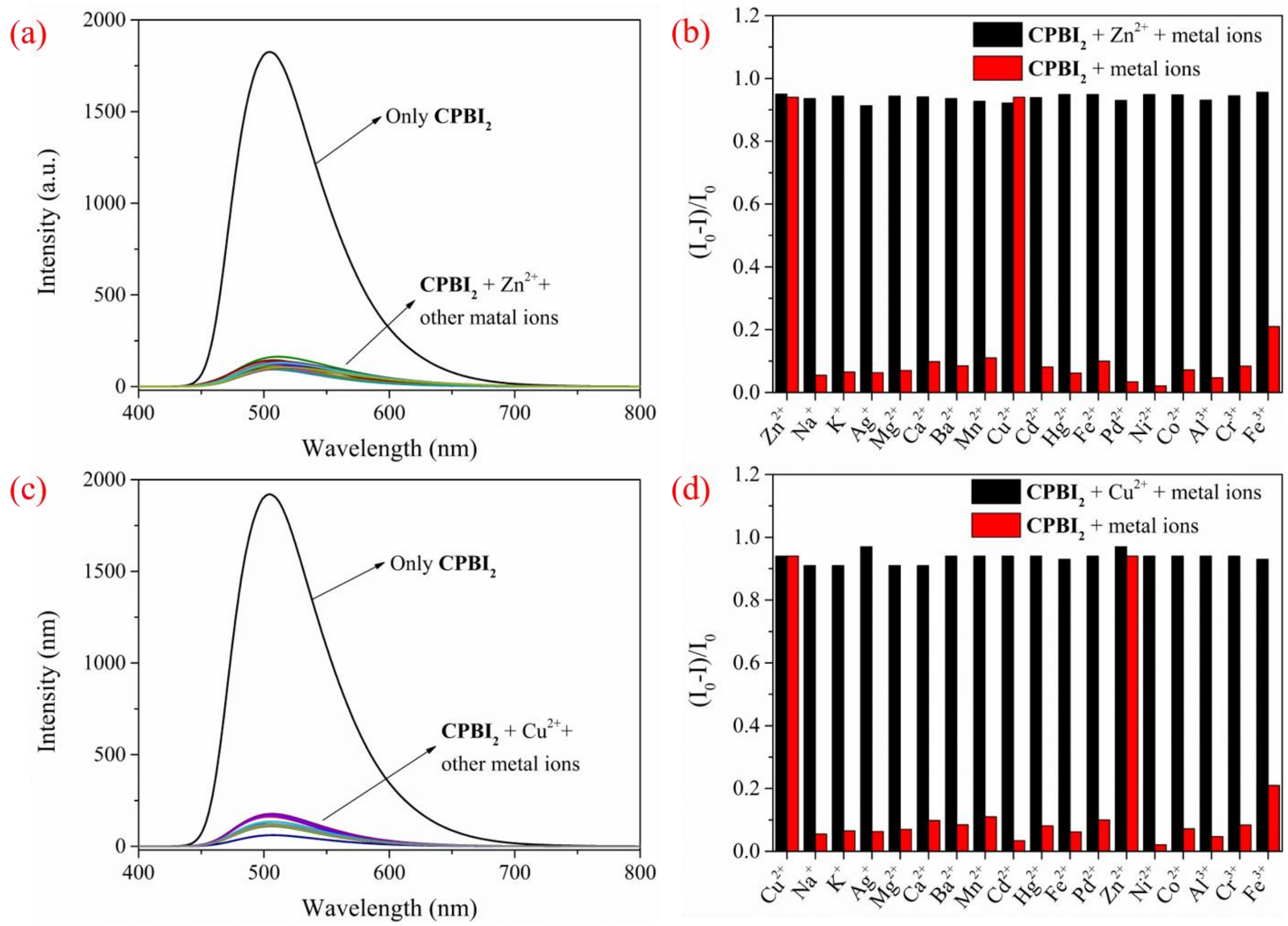

Figure S15. Fluorescence emission spectra of sensor **CPBI₂** solution (1 mg/15 mL in DMSO/H_2O, v/v, 9/1) with Zn^{2+} (100 μM) and other metal ions (100 μM) (a) and comparison of fluorescence quenching rate (b), fluorescence emission spectra of Cu^{2+} (100 μM) and other metal ions (c) and comparison of fluorescence quenching rate (d), λ_{ex} = 375 nm.

14. The selective experiments of $CPBI_1$ towards metal ions

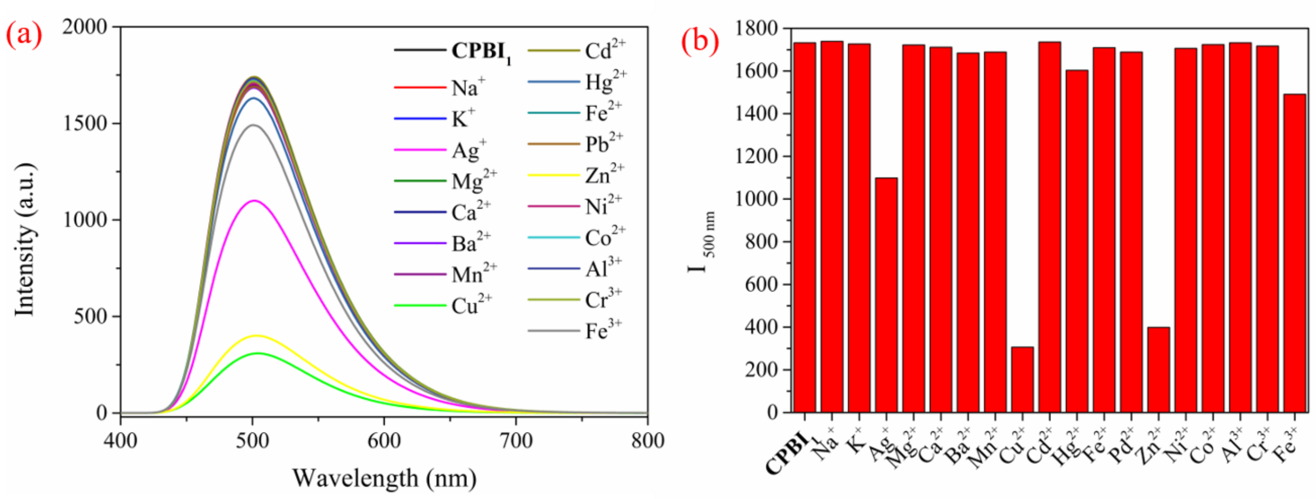

Figure S16. The fluorescence selectivity study of different metal ions (100 μM) in **$CPBI_1$** (1 mg/15 mL in DMSO/H_2O, v/v, 9/1) solution (a), the influence of adding different metal ions (100 μM) on **$CPBI_1$** fluorescence intensity at 500 nm (b), λ_{ex} = 375 nm.

15. The competitive experiments of $CPBI_1$ for Cu^{2+} or Zn^{2+}

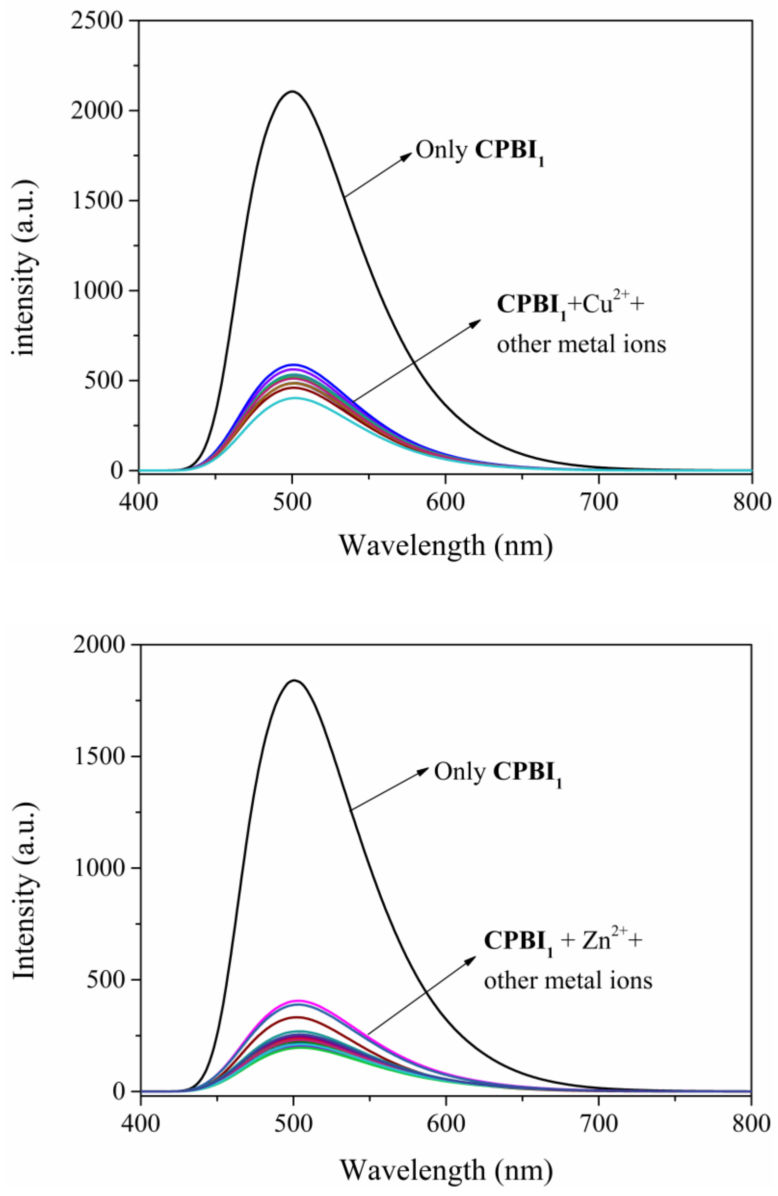

Figure S17. Fluorescence emission spectra of sensor **CPBI₁** solution (1 mg/15 mL in DMSO/H_2O, v/v, 9/1) with Cu^{2+} (100 μM) and other metal ions (100 μM) (the upper), fluorescence emission spectra of Zn^{2+} (100 μM) and other metal ions (100 μM) (the lower), λ_{ex} = 375 nm.

16. Fluorescent titration and detection limit of $CPBI_1$ for Cu^{2+}

Based on the fluorescence titration data of $\mathbf{CPBI_1}$ and Cu^{2+} (**Figure S18**), the LOD of $\mathbf{CPBI_1}$ for Cu^{2+} is obtained as 8.2×10^{-9} M according to the LOD calculation formula "LOD = 3δ/K".

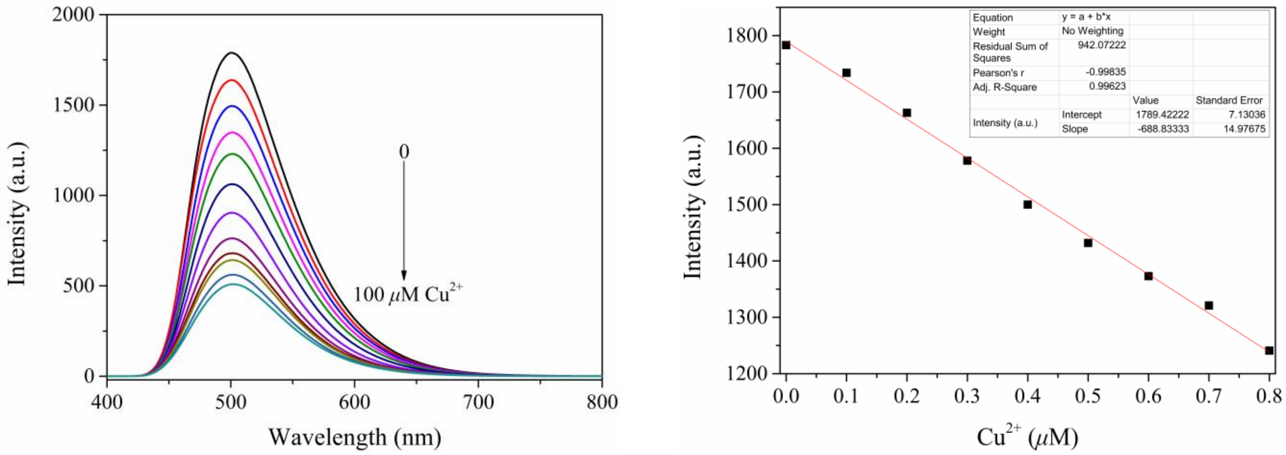

Figure S18. Fluorescence emission spectra of $\mathbf{CPBI_1}$ (1 mg/15 mL in DMSO/H_2O, v/v, 9/1) solution with different concentrations of Cu^{2+} and the linear relationship between $\mathbf{CPBI_1}$ and low concentrations of Cu^{2+}, λ_{ex} = 375 nm.

Compared with Cu^{2+} sensors reported recently, $\mathbf{CPBI_1}$ can sensitively detect Cu^{2+} (more comparison can be seen in the following **Table S5** in this **SM**).

17. Fluorescent titration and detection limit of **$CPBI_1$** for Zn^{2+}

Based on the fluorescence titration data of **$CPBI_1$** and Zn^{2+} (**Figure S19**), the LOD of **$CPBI_1$** for Zn^{2+} is obtained as 8.51×10^{-9} M according to the LOD calculation formula "LOD = $3\delta/K$".

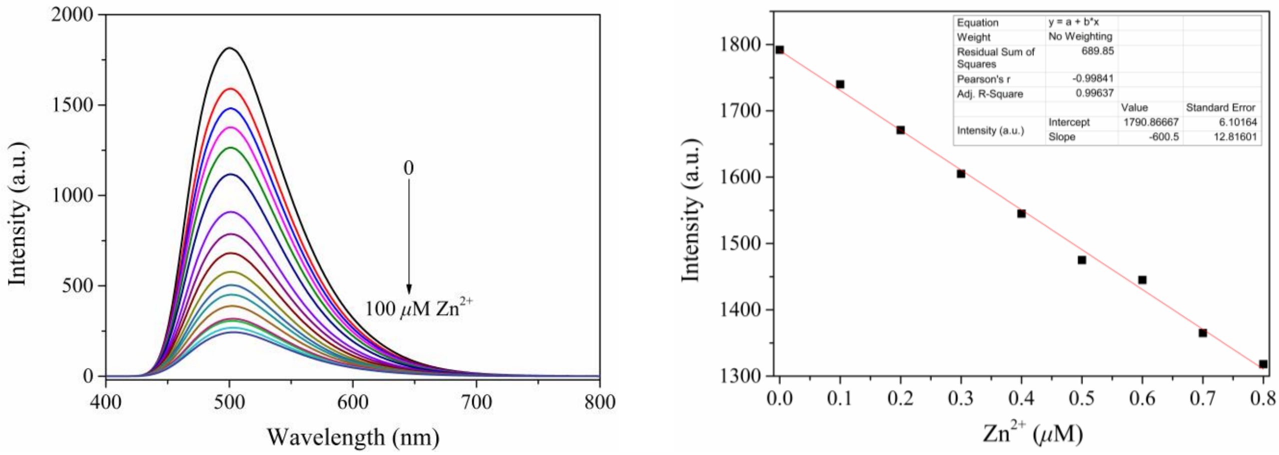

Figure S19. Fluorescence emission spectra of **$CPBI_1$** (1 mg/15 mL in DMSO/H_2O, v/v, 9/1) solution with different concentrations of Zn^{2+} and the linear relationship between **$CPBI_1$** and low concentrations of Zn^{2+}, λ_{ex} = 375 nm.

Compared with Zn^{2+} sensors reported recently, **$CPBI_1$** can sensitively detect Zn^{2+} (more comparison can be seen in the following **Table S6** in this **SM**).

18. The selective experiments of $CPBI_3$ towards metal ions

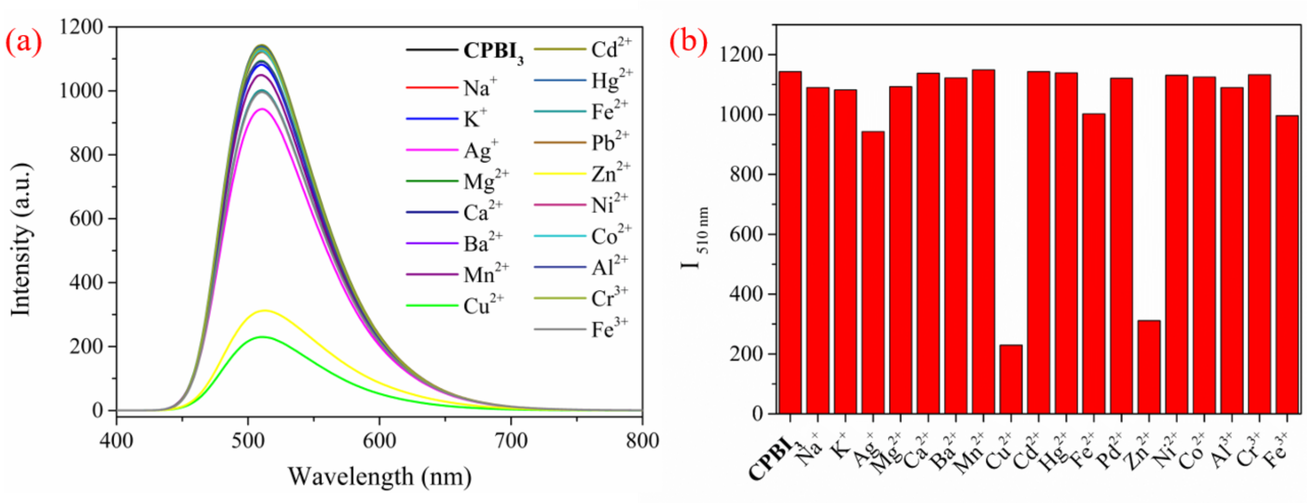

Figure S20. The fluorescence selectivity study of different metal ions (100 μM) in $\mathbf{CPBI_3}$ (1 mg/15 mL in DMSO/H_2O, v/v, 9/1) solution (a), the influence of adding different metal ions (100 μM) on $\mathbf{CPBI_3}$ fluorescence intensity at 510 nm (b), λ_{ex} = 375 nm.

19. The competitive experiments of $CPBI_3$ for Cu^{2+} or Zn^{2+}

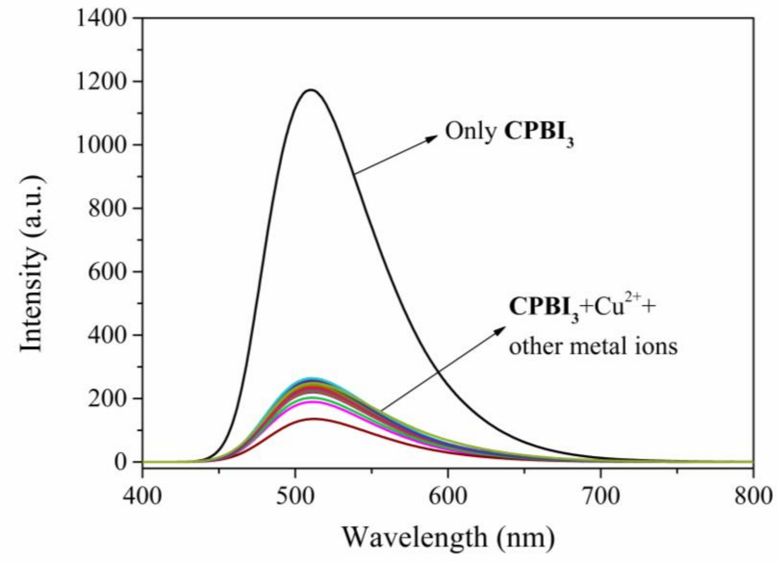

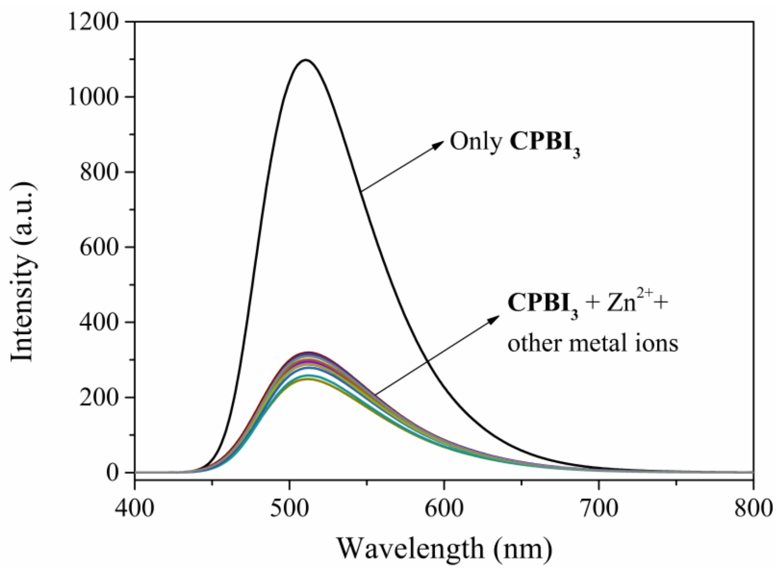

Figure S21. Fluorescence emission spectra of sensor **$CPBI_3$** solution (1 mg/15 mL in DMSO/H_2O, v/v, 9/1) with Cu^{2+} (100 μM) and other metal ions (100 μM) (the upper), fluorescence emission spectra of Zn^{2+} (100 μM) and other metal ions (100 μM) (the lower), λ_{ex} = 375 nm.

20. Fluorescent titration and detection limit of CPBI$_3$ for Cu^{2+}

Based on the fluorescence titration data of **CPBI$_3$** and Cu^{2+} (**Figure S22**), the LOD of **CPBI$_3$** for Cu^{2+} is obtained as 4.42×10^{-9} M according to the LOD calculation formula "LOD = $3\delta/K$".

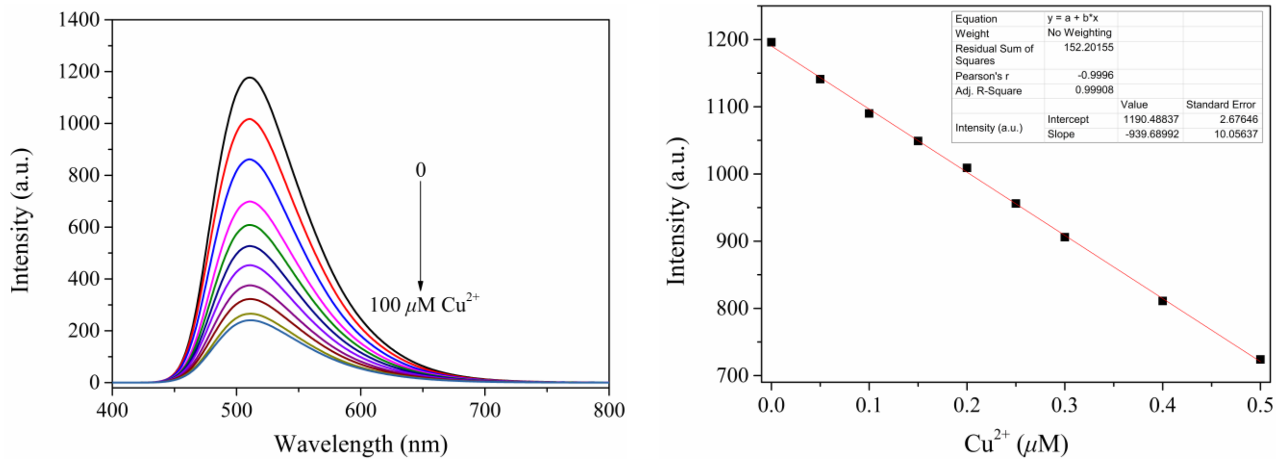

Figure S22. Fluorescence emission spectra of **CPBI$_3$** (1 mg/15 mL in DMSO/H_2O, v/v, 9/1) solution with different concentrations of Cu^{2+} and the linear relationship between **CPBI$_3$** and low concentrations of Cu^{2+}, λ_{ex} = 375 nm.

Compared with Cu^{2+} sensors reported recently, **CPBI$_3$** can sensitively detect Cu^{2+} (more comparison can be seen in the following **Table S5** in this **SM**).

21. Fluorescent titration and detection limit of $CPBI_3$ for Zn^{2+}

Based on the fluorescence titration data of **$CPBI_3$** and Zn^{2+} (**Figure S23**), the LOD of **$CPBI_3$** for Zn^{2+} is obtained as 3.68×10^{-9} M according to the LOD calculation formula "LOD = $3\delta/K$".

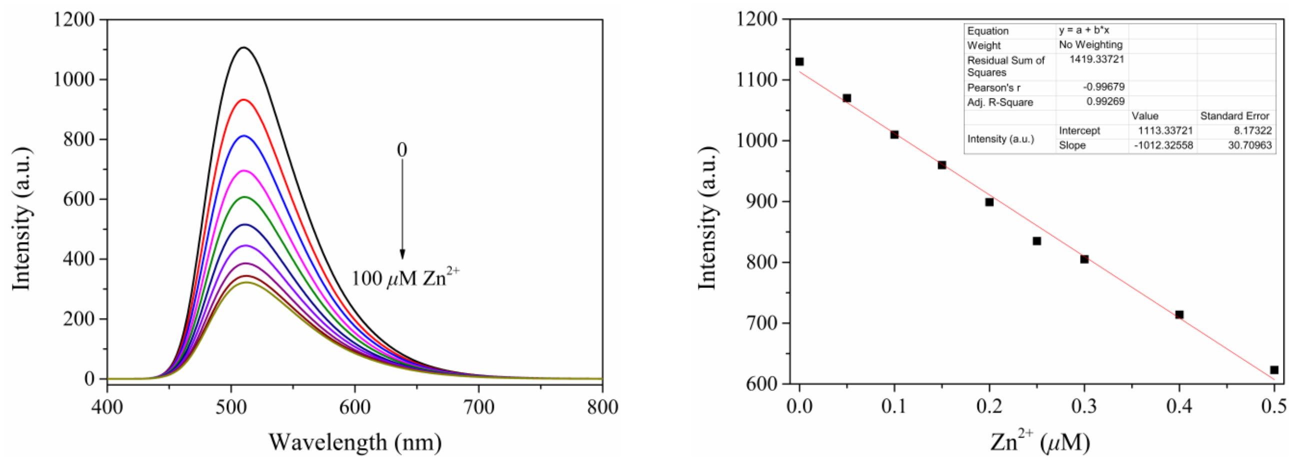

Figure S23. Fluorescence emission spectra of **$CPBI_3$** (1 mg/15 mL in DMSO/H_2O, v/v, 9/1) solution with different concentrations of Zn^{2+} and the linear relationship between **$CPBI_3$** and low concentrations of Zn^{2+}, λ_{ex} = 375 nm.

Compared with Zn^{2+} sensors reported recently, **$CPBI_3$** can sensitively detect Zn^{2+} (more comparison can be seen in the following **Table S6** in this **SM**).

22. The selective experiments of CPBI$_4$ towards metal ions

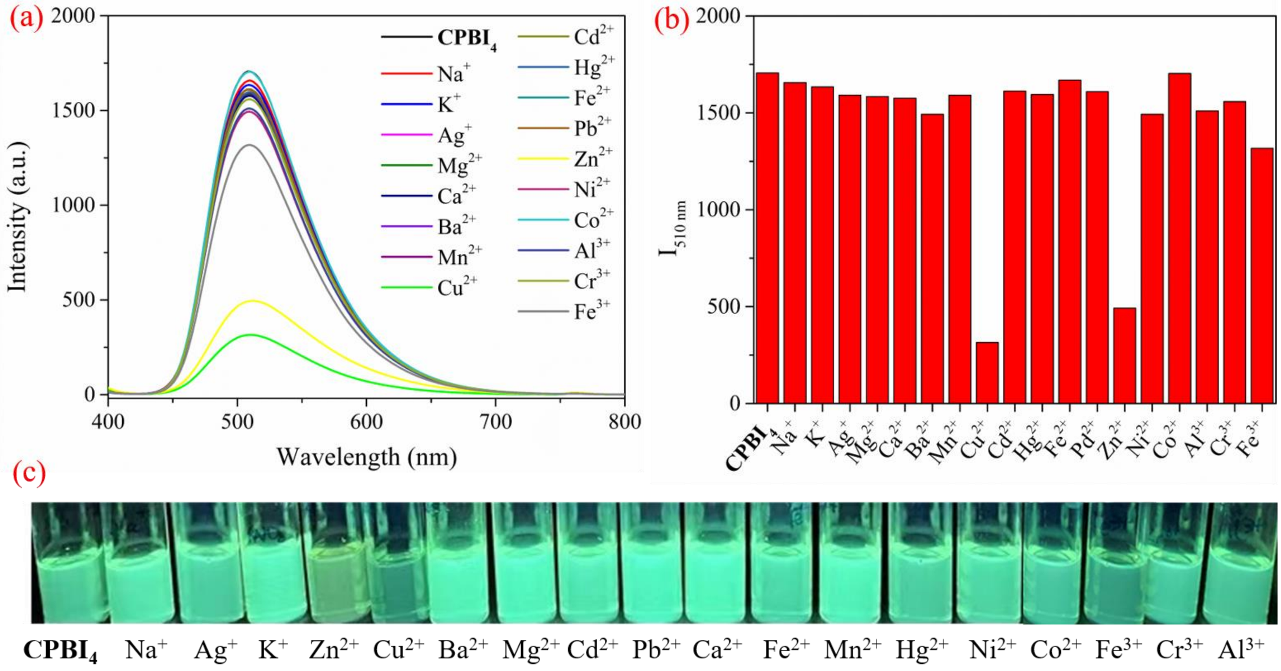

Figure S24. The fluorescence selectivity study of different metal ions (100 μM) in **CPBI$_4$** (1 mg/15 mL in DMSO/H_2O, v/v, 9/1) solution, λ_{ex} = 375 nm (a), the influence of adding different metal ions (100 μM) on **CPBI$_4$** fluorescence intensity at 510 nm (b) and the color change of the solution (c).

23. The competitive experiments of $CPBI_4$ for Cu^{2+} or Zn^{2+}

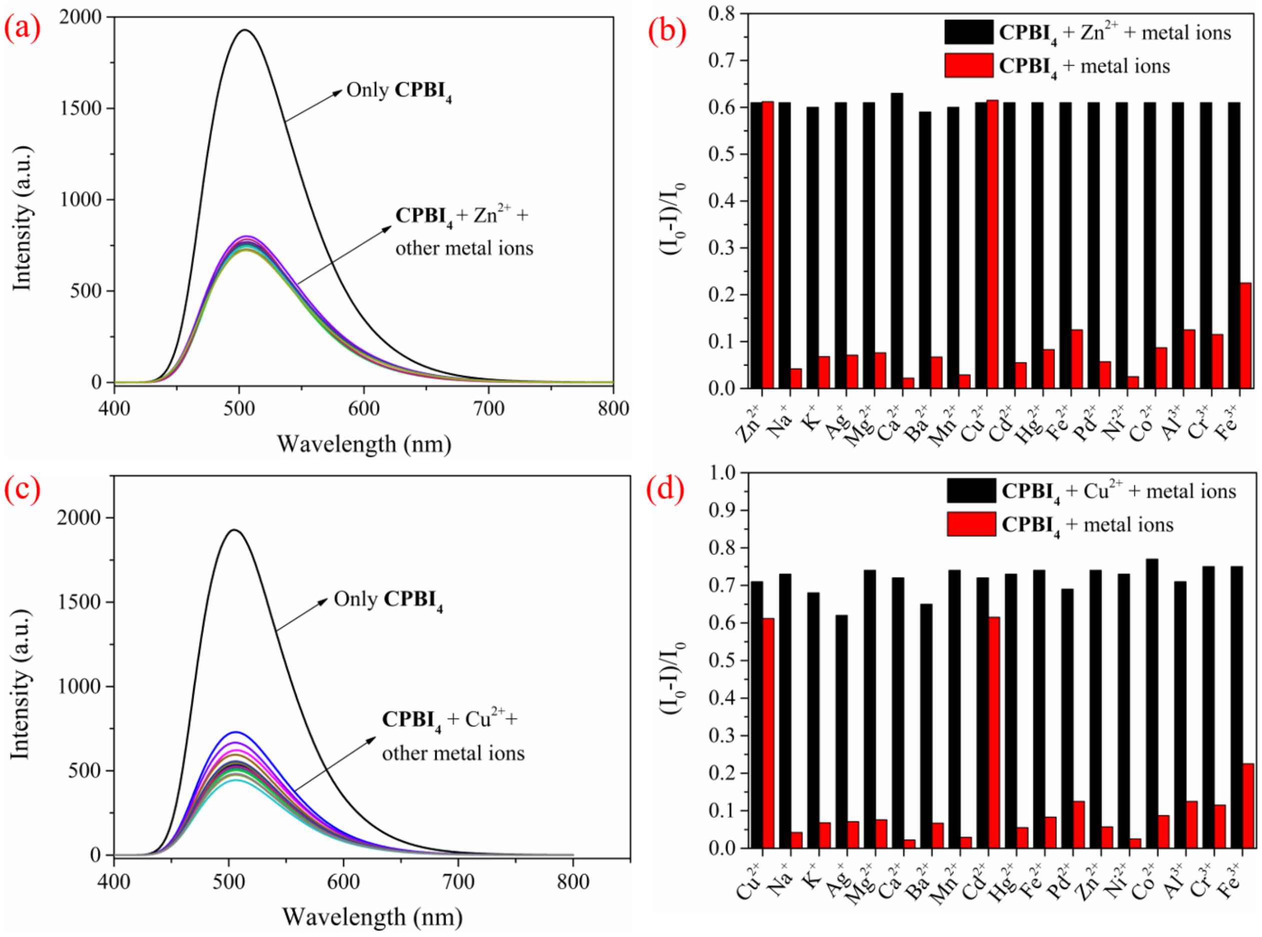

Figure S25. Fluorescence emission spectra of sensor **$CPBI_4$** solution (1 mg/15 mL in DMSO/H_2O, v/v, 9/1) with Zn^{2+} (100 μM) and other metal ions (100 μM) (a) and comparison of fluorescence quenching rate (b), fluorescence emission spectra of Cu^{2+} (100 μM) and other metal ions (100 μM) (c) and comparison of fluorescence quenching rate (d), λ_{ex} = 375 nm.

24. Fluorescent titration and detection limit of $CPBI_4$ for Cu^{2+}

Based on the fluorescence titration data of **$CPBI_4$** and Cu^{2+} (**Figure S26**), the LOD of **$CPBI_4$** for Cu^{2+} is obtained as 2.2×10^{-9} M according to the LOD calculation formula "LOD = $3\delta/K$".

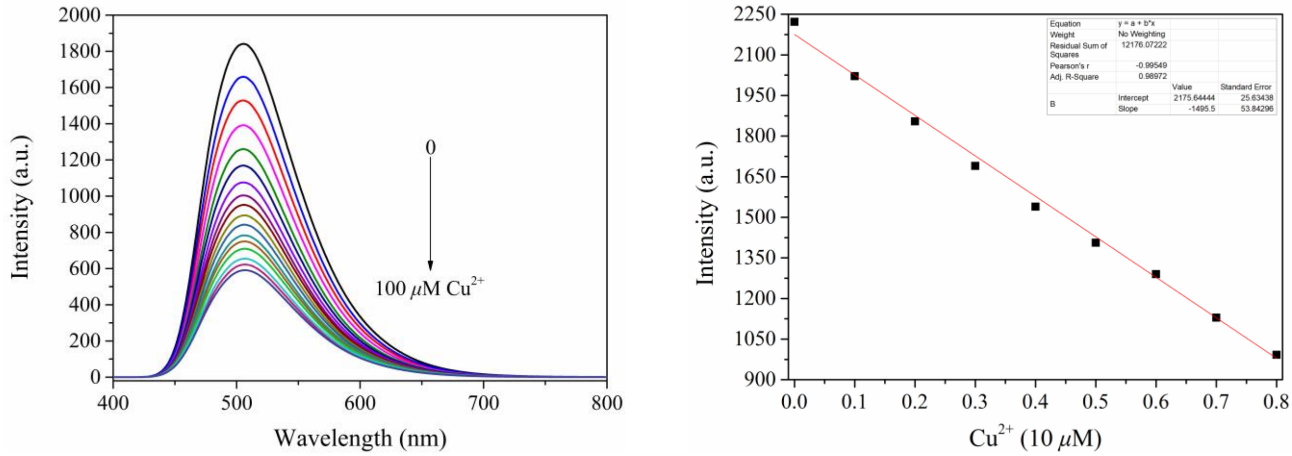

Figure S26. Fluorescence emission spectra of **$CPBI_4$** (1 mg/15 mL in DMSO/H_2O, v/v, 9/1) solution with different concentrations of Cu^{2+} and the linear relationship between **$CPBI_4$** and low concentrations of Cu^{2+}, λ_{ex} = 375 nm.

Compared with Cu^{2+} sensors reported recently, **$CPBI_4$** can sensitively detect Cu^{2+} (more comparison can be seen in the following **Table S5** in this **SM**).

25. Fluorescent titration and detection limit of $CPBI_4$ for Zn^{2+}

Based on the fluorescence titration data of **$CPBI_4$** and Zn^{2+} (**Figure S27**), the LOD of **$CPBI_4$** for Zn^{2+} is obtained as 1.93×10^{-9} M according to the LOD calculation formula "LOD = $3\delta/K$".

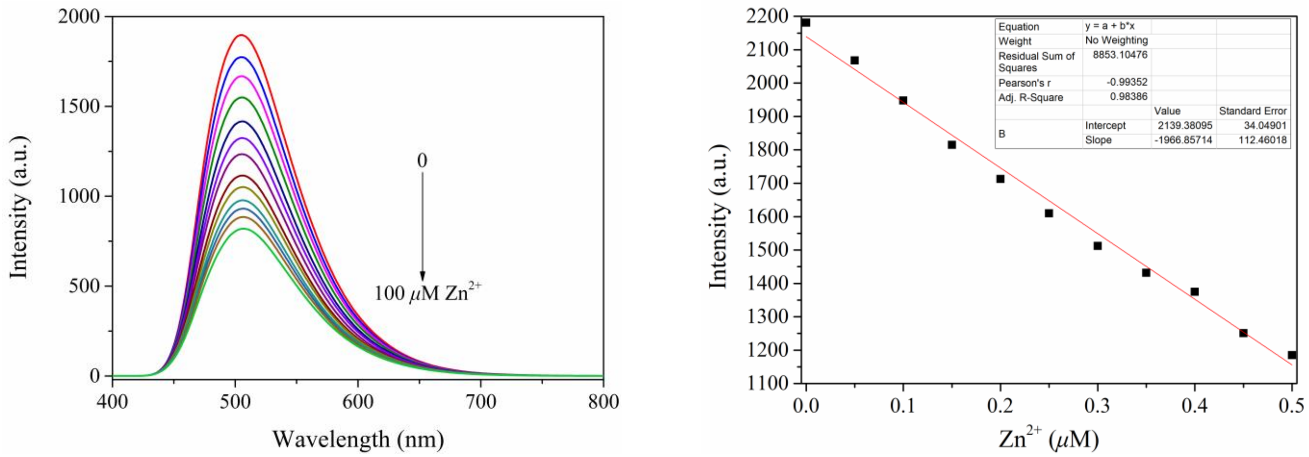

Figure S27. Fluorescence emission spectra of **$CPBI_4$** (1 mg/15 mL in DMSO/H_2O, v/v, 9/1) solution with different concentrations of Zn^{2+} and the linear relationship between **$CPBI_4$** and low concentrations of Zn^{2+}, λ_{ex} = 375 nm.

Compared with Zn^{2+} sensors reported recently, **$CPBI_4$** can sensitively detect Zn^{2+} (more comparison can be seen in the following **Table S6** in this **SM**).

26. The plots of **CPBI4** *vs* concentration of Cu^{2+} and Zn^{2+}

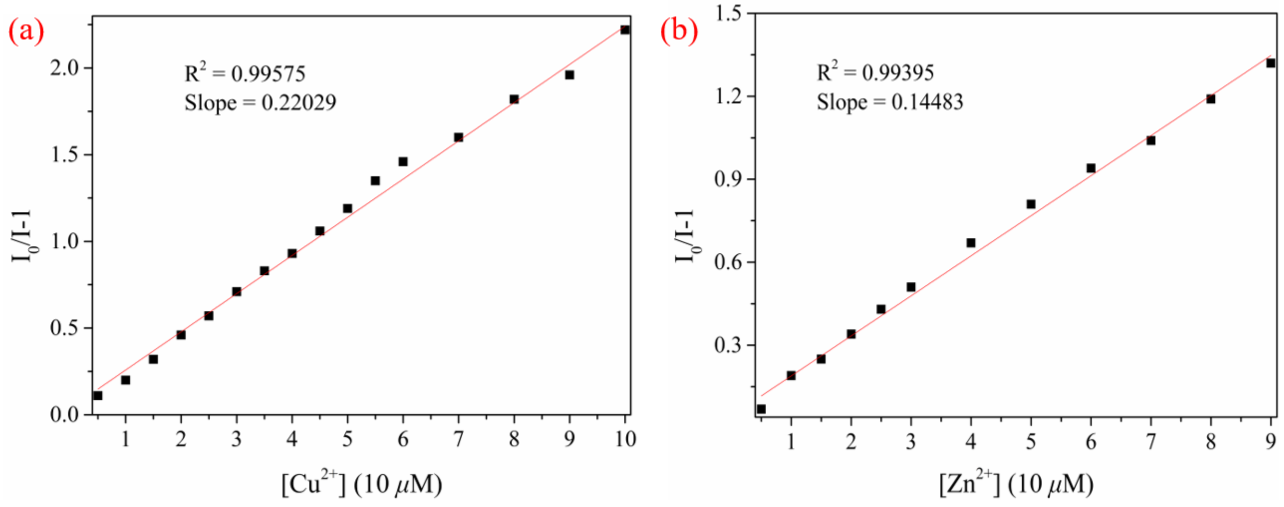

Figure S28. Stern-Volmer diagram of the interaction of sensors **CPBI4** with Cu^{2+} (a) and Zn^{2+} (b) (the illustration shows the Stern-Volmer linear diagram at low concentrations).

27. Comparison of detection limits of Cu^{2+} and Zn^{2+} by serial **CPBI*n***

Table S4. Comparison of LOD when the sensor **CPBI*n*** detects Cu^{2+} and Zn^{2+}.

Sensor	LOD for Cu^{2+}	LOD for Zn^{2+}
**CPBI1**	8.20×10^{-9} M	8.51×10^{-9} M
**CPBI2**	5.98×10^{-9} M	6.02×10^{-9} M
**CPBI3**	4.42×10^{-9} M	3.68×10^{-9} M
**CPBI4**	2.21×10^{-9} M	1.93×10^{-9} M

28. The changes of FT-IR spectra and morphology before or after the combination with analytes

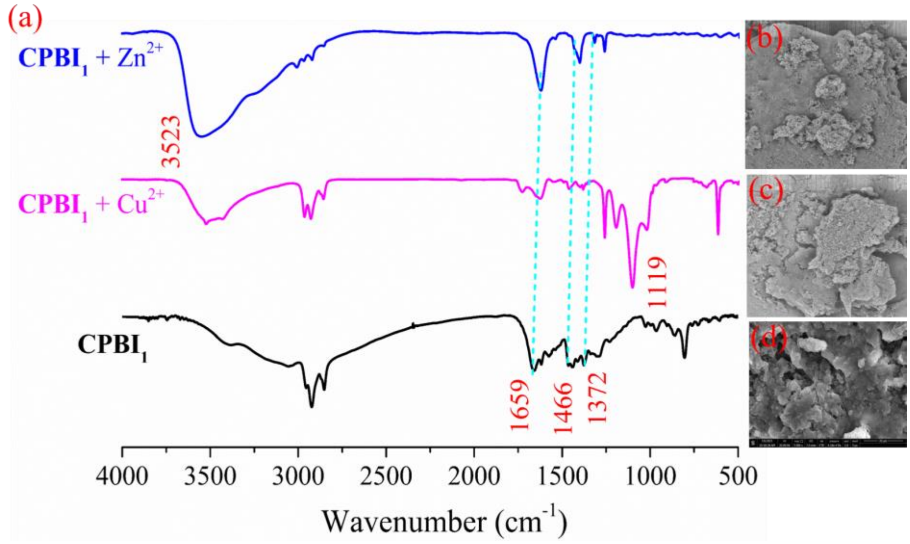

Figure S29. FT-IR spectra (a), SEM images of **$CPBI_1$** before and after the addition of Cu^{2+} or Zn^{2+} (b: **$CPBI_1$** + Zn^{2+}; c: **$CPBI_1$** + Cu^{2+}; d: **$CPBI_1$**).

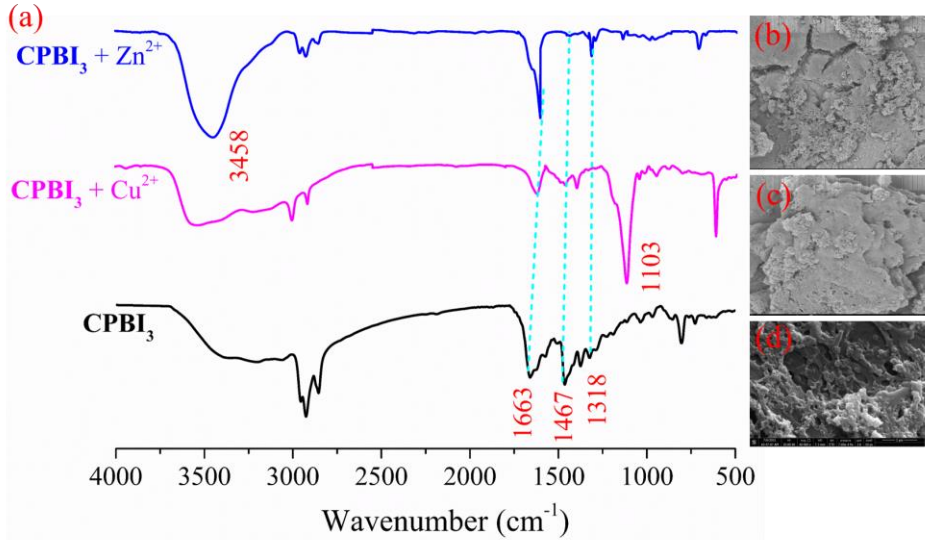

Figure S30. FT-IR spectra (a), SEM images of **$CPBI_3$** before and after the addition of Cu^{2+} or Zn^{2+} (b: **$CPBI_3$** + Zn^{2+}; c: **$CPBI_3$** + Cu^{2+}; d: **$CPBI_3$**).

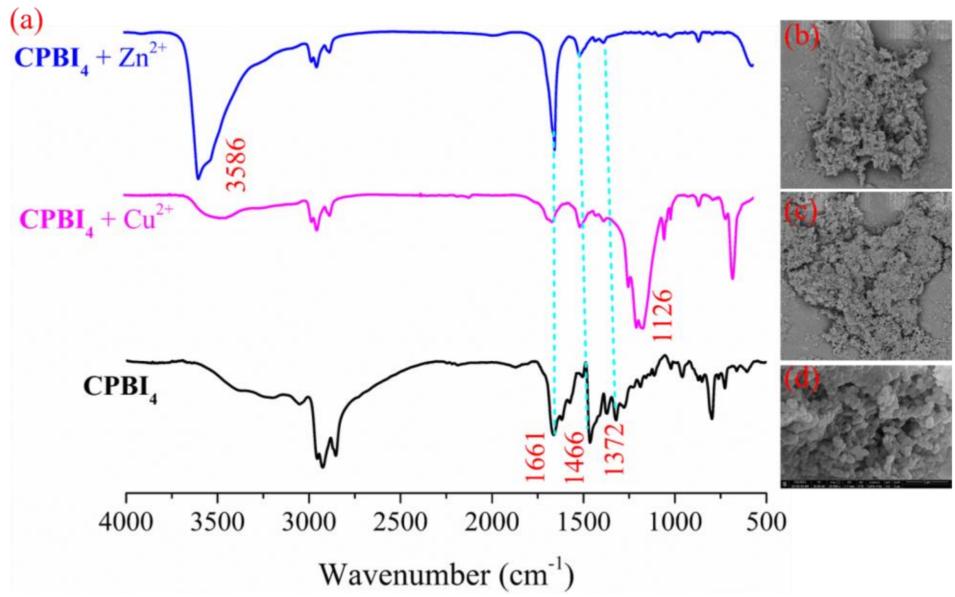

Figure S31. FT-IR spectra (a), SEM images of **CPBI4** before and after the addition of Cu^{2+} or Zn^{2+} (b: **CPBI4** + Zn^{2+}; c: **CPBI4** + Cu^{2+}; d: **CPBI4**).

29. TCSPC plots for CPBI2 and CPBI4 interacted with Cu^{2+} and Zn^{2+}

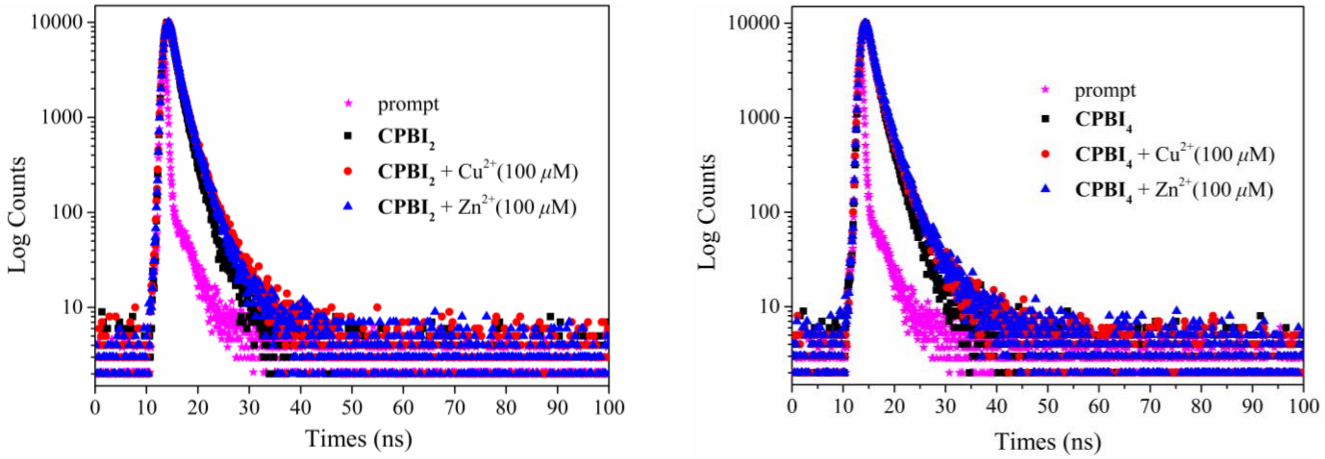

Figure S32. Time-correlated single photon counting (TCSPC) plot for **CPBI2** (left) and **CPBI4** (right) interacted with Cu^{2+} and Zn^{2+} (λ_{ex} = 375 nm, λ_{em} = 504 nm for **CPBI2** and λ_{em} = 510 nm for **CPBI4**, respectively).

30. Comparison of $CPBI_n$ with Cu^{2+} and Zn^{2+} probes available in the literature

Table S5. The comparison of probe **$CPBI_n$** with the reported Cu^{2+} probes in solution.

No.	Probe type	Solvent	Detection limit	Signal types	Ref.
1	In-MOF based dual-responsive fluorescence probe	Aqueous solution	6.06×10^{-8} M	Turn-off	[14]
2	Rhodamine B integrated ZIF-8	Aqueous solution	1.91×10^{-7} M	Fluorescence ratiometric	[15]
3	Possessing an anthracene scaffold as a fluorophore and ferrocene as a redox active unit	Aqueous media	3.9×10^{-8} M	Turn-off	[16]
4	$Fe_3O_4@SiO_2-NH_2$/CQDs	Aqueous solution	1.6×10^{-7} M	Turn-off	[12]
5	A dicyanomethylene-4H-pyran-based	PBS solution (10 mM, pH = 7.4, 50% DMSO)	2.54×10^{-8} M	Turn-on	[17]
6	N-acetyl-L-cysteines (NALC) (NALC-CdS QDs)	Aqueous solution	4.8×10^{-7} M	Turn-off	[18]
7	Dual-functional peptide-based probe	Aqueous solution	7.67×10^{-8} M	Turn off	[19]
8	4-Hydroxyhypoxidone	Aqueous solution	4.0×10^{-5} M	Turn-on	[10]
9	Dibenzimidazole-based	Aqueous solution	9.4×10^{-8} M	Turn off	[21]
10	BODIPY derivatives	MeOH	1.0×10^{-7} M	Turn-off	[22]
11	Carbon dots	EtOH /PBS buffer (1:4, *V/V*, 10 mM, pH = 7.4, 37 °C)	2.89×10^{-9} M	Turn-off	[23]
12	A hemicyanine scaffold as the fluorophore	PBS buffer (1% DMSO, pH = 7.4)	4.0×10^{-9} M	Turn-on	[24]
13	Carbazole-based	Aqueous solution	7.80×10^{-6} M	Turn-off	[25]
14	Aminophenylbenzimidazole derivative	Aqueous HEPES buffer (10 mM, pH = 7.4)	2.3×10^{-8} M	Ratiometric	[26]
15	Pyrene-based	DMSO/H_2O (2:8 *V/V*), 50 mM HEPES, pH = 7.4)	7.3×10^{-10} M	Turn-off	[27]

16	A coumarin-containing Schiff base	THF/H_2O (1/99, *V/V*)	3.6 × 10^{-7} M	Turn-off	[28]
17	Carbon nitride@gold nanoclusters (WS-GCN@AuNCs) nanocomposite	Aqueous solution	3.63 × 10^{-9} M	Turn-off	[13]
18	Oevinyl protected hydroxyl benzaldehyde	CH_3CN-PBS buffer (3:7, *V/V*)	6.7 × 10^{-7} M	Ratiometric	[29]
19	A dansyl derivative	HEPES buffer solution (10 mM, pH = 7.4)	2.9 × 10^{-7} M	Turn-off	[30]
20	[1,2-Phenylenediamine-2,6-pyridinedialdehyde macrocyclic Schiff base] (BP-MSB) based on 2,6-pyridinedialdehyde	Aqueous solution	8.3 × 10^{-10} M	Turn-off	[31]
This work	**$CPBI_1$**	DMSO/H_2O (9/1, *V/V*)	8.20 × 10^{-9} M	Turn-off	
	**$CPBI_2$**	DMSO/H_2O (9/1, *V/V*)	5.98 × 10^{-9} M	Turn-off	
	**$CPBI_3$**	DMSO/H_2O (9/1, *V/V*)	4.42 × 10^{-9} M	Turn-off	
	**$CPBI_4$**	DMSO/H_2O (9/1, *V/V*)	2.21 × 10^{-9} M	Turn-off	

Table S6. The comparison of probe **CPBI*n*** with the reported Zn^{2+} probes in solution.

No.	Probe type	Solvent	Detection limit	Signal types	Ref.
1	Quinoline-based chemosensor	Aqueous solution	6.0 × 10^{-7} M	Turn-on	[18]
2	Schiff base	H_2O/CH_3OH (80/20, *V*/*V*)	3.84 × 10^{-8}M	Turn-on	[32]
3	5,5′′-Diaryl-2,2′:6′,2′′-terpyridines	CH_3CN (10^{-5} M)	1.0 × 10^{-10} M	Turn-on	[33]
4	7,8-Benzochromone-3-carbaldehyde(fluorescein)-hydrazone	EtOH/H_2O (9/1, *V*/*V*)	3.4 × 10^{-7} M	Turn-on	[34]
5	Anthracene possessing amide functionality	CH_3CN	3.03 × 10^{-6} M	Turn-on	[35]
6	Based rhodamine 6G condensed isophorone moiety (RHI)	EtOH/H_2O (8/2, *V*/*V*)	8.22 × 10^{-8} M	Turn-on	[36]
7	(E)-1-(6-(4-(2-(2-(2-methoxy-ethoxy)ethoxy)eth-oxy)-3,5-di-methylstyryl)quinolin-2-yl)-*N*,*N*-bis(pyridin-2-ylmethyl)meth-anamine (TEO-MPVQ)	Methanol/Buffer (1/9, *V*/*V*)	2.5 × 10^{-8} M	Turn-on	[37]
8	Naphthalimide-derived	HEPES	1.0 × 10^{-5} M	Turn-on	[38]
9	A star-shaped Schiff base	Aqueous solution	2.4 × 10^{-6} M	Turn-on	[39]
10	8-Amidoquinoline Derivatives	Aqueous solution	3.36 × 10^{-8} M	Turn-on	[40]
11	Naphthalene derivative	EtOH/H_2O (9/1, *V*/*V*, pH = 7.4)	3.17 × 10^{-9} M	Turn-on	[41]
12	A bisphenol based	DMSO/H_2O (1/9, *V*/*V*)	2.8 × 10^{-10} M	Turn-on	[42]
13	Formylcoumarin and aminoquinoline moieties	MeOH-H_2O (4/1, *V*/*V*)	3.75 × 10^{-8} M	Turn-on	[43]
14	A coumarin-based	PBS (pH = 7.24, 60% DMF)	9.5 × 10^{-7} M	Turn-on	[44]
15	Molecular brushes of poly(2-oxazoline)	Aqueous solution	8.9 × 10^{-6} M	Turn-on	[45]
16	Anthracene-based	DMSO/H_2O (3:1, *V*/*V*)	3.6 × 10^{-8} M	Turn-on	[46]
17	A novel salen derivative	DMF	1.63 × 10^{-8} M	Turn-on	[47]

18	Heterocyclic-fused naphthal-imide fluorophore, and the zinc receptor, *N*,*N*-bis(2-pyridyl-methyl)-ethylenediamine (BPEN).	Phosphate buffer solution	4.0×10^{-9} M	Turn-on	[54]
19	Thiophen-based	CH_3CN	6.0×10^{-7} M	Turn-on	[48]
20	Benzothiazole as signal unit	DMF/H_2O (1/1, *V*/*V*, 0.01 M HEPES, pH = 6.0)	1.27×10^{-7} M	Turn-on	[49]
This work	**CPBI$_1$**	DMSO/H_2O (9/1, *V*/*V*)	8.51×10^{-9} M	Turn-off	
	**CPBI$_2$**	DMSO/H_2O (9/1, *V*/*V*)	6.02×10^{-9} M	Turn-off	
	**CPBI$_3$**	DMSO/H_2O (9/1, *V*/*V*)	3.68×10^{-9} M	Turn-off	
	**CPBI$_4$**	DMSO/H_2O (9/1, *V*/*V*)	1.93×10^{-9} M	Turn-off	

References

[1] Huang, F.; Pingitore, A.T.; Benicewicz, B.C. Electrochemical hydrogen separation from reformate using high temperature polybenzimidazole (PBI) membranes: the role of chemistry. *ACS Sustainable Chem. Eng.* **2020**, *8*, 6234-6242.

[2] Konovalova, A.; Stock, D.; Schroder, S.; Park, H.S.; Jang, J.H.; Kim, H.J.; Han. J.; Schroder, D.; Henkensmeier, D. Partially methylated polybenzimidazoles as coating for alkaline zinc anodes. *J. Membr. Sci.* **2020**, *610*, 118254.

[3] Aili, D.; Yang, J.S.; Jankova, K.; Henkensmeier, D.; Li, Q.F. From polybenzimidazoles to poly-benzimidazoliums and polybenzimidazolides. *J. Mater. Chem. A* **2020**, *8*, 12854-12886.

[4] Hussaini, S.Y.; Haque, R.A.; Fatima, T.; Agha, T.M.; Majid, A.M.S.A.; Abdallah, H.H.; Razali, M.R. Nitrile functionalized silver(I) *N*-heterocyclic carbene complexes: DFT calculations and anti-tumor studies. *Transition Met. Chem.* **2018**, *43*, 301-312.

[5] Shannon, M.S.; Hindman, M.S.; Danielsen, S.P.O.; Tedstone, J.M.; Gilmore, R.D.; Bara, J.E. Properties of alkyl benzimidazoles for CO_2 and SO_2 capture and comparisons to ionic liquids. *Sci. China: Chem.* **2012**, *55*, 1638-1647.

[6] Shahini, C.; Achar, G.; Budagumpi, S.; Tacke, M.; Patil, S.A. Synthesis, structural investigation and antibacterial studies of non-symmetrically *p*-nitrobenzyl substituted benzimidazole *N*-hetero-cyclic carbene-silver(I) complexes. *Inorg. Chim. Acta* **2017**, *466*, 432-441.

[7] Kaewnok, N.; Sirirak, J.; Jungsuttiwong, S.; Wongnongwa, Y.; Kamkaew, A.; Petdum, A.; Panchan, W.; Sahasithiwat, S.; Sooksimuang, T.; Charoenpanich, A.; Wanichacheva, N. Detection of hazardous mercury ion using [5]helicene-based fluorescence probe with “turn-on” sensing response for practical applications. *J. Hazard. Mater.* **2021**, *418*, 126242.

[8] He, Y.L.; Li, N.; Li, W.K.; Zhang, X.X.; Zhang, X.; Liu, Z.X.; Liu, Q.Y. 5,10,15,20-Tetrakis (4-carboxylphenyl) porphyrin functionalized $NiCo_2S_4$ yolk-shell nanospheres: Excellent peroxidase-like activity, catalytic mechanism and fast cascade colorimetric biosensor for cholesterol. *Sens. Actuators, B* **2021**, *326*, 128850.

[9] Giri, D.; Bankura, A.; Patra, S.K. Poly(benzodithieno-imidazole-*alt*-carbazole) based pi-conju-gated copolymers: Highly selective and sensitive turn-off fluorescent probes for Hg^{2+}. *Polymer* **2018**, *158*, 338-353.

[10] Li, W.T.; Zhang, X.N.; Hu, X.T.; Shi, Y.Q.; Li, Z.H.; Huang, X.W.; Zhang, W.; Zhang, D.; Zou, X.B.; Shi, J.Y. A smartphone-integrated ratiometric fluorescence sensor for visual detection of cadmium ions. *J. Hazard. Mater.* **2021**, *408*, 124872.

[11] Du, Y.; Song, Y.H.; Hao, J.; Cai, K.Y.; Liu, N.; Yang, L.; Wang, L. Ratiometric fluorescence detection of O^{-2} (center dot-) based on dual-emission schiff base polymer/rhodamine-B nano-composites. *Talanta* **2019**, *198*, 316-322.

[12] Dong, S.L.; Wang, S.N.; Wang, X.L.; Zhai, L.Z. Superparamagnetic nanocomposite Fe_3O_4@SiO_2-NH_2/CQDs as fluorescent probe for copper (II) detection. *Mater. Lett.* **2020**, *278*, 128404.

[13] Guo, X.R.; Huang, J.Z.; Wang, M.; Wang, L.S. A dual-emission water-soluble g-C_3N_4@AuNCs-based fluorescent probe for label-free and sensitive analysis of trace amounts of ferrous (II) and copper (II) ions. *Sens. Actuators, B* **2020**, *309*, 127766.

[14] Guan, B.-B.; Li, Q.; Xu, Y.-T.; Chen, L.-H.; Wu, Z.-S.; Fan, Z.-L.; Zhu, W. Highly selective and sensitive detection towards cationic Cu^{2+} and Fe^{3+} contaminants via an In-MOF based dual-responsive fluorescence probe. *Inorg. Chem. Commun.* **2020**, *122*, 108273.

[15] Du, T.; Wang, J.; Zhang, T.S.; Zhang, L.; Yang, C.Y.; Yue, T.L.; Sun, J.; Li, T.; Zhou, M.G.; Wang, J.L. An integrating platform of ratiometric fluorescent adsorbent for unconventional real-time removing and monitoring of copper ions. *ACS Appl. Mater. Interfaces* **2020**, *12*, 13189-13199.

[16] Karakus, E.; Gunduz, S.; Liv, L.; Ozturk, T. Fluorescent and electrochemical detection of Cu (II) ions in aqueous environment by a novel, simple and readily available AIE probe. *J. Photochem. Photobiol., A* **2020**, *400*, 112702.

[17] Li, Z.; Xu, Y.Q.; Xu, H.D.; Cui, M.Y.; Liu, T.G.; Ren, X.Y.; Sun, J.F.; Deng, D.W.; Gu, Y.Q.; Wang, P. A dicyanomethylene-4H-pyran-based fluorescence probe with high selectivity and sensitivity for detecting copper (II) and its bioimaging in living cells and tissue. *Spectrochim. Acta, Part A* **2021**, *244*, 118819.

[18] Zhao, R.J.; Wang, Z.Z.; Tian, X.; Shu, H.; Yang, Y.; Xiao, X.C.; Wang, Y.D. Excellent fluorescence detection of Cu^{2+} in water system using *N*-acetyl-L-cysteines modified CdS quantum dots as fluorescence probe. *Nanotechnol.* **2021**, *32*, 405707.

[19] Wang, P.; Sun, L.Y.; Wu, J.; Yang, X.P.; Lin, P.C.; Wang, M. A dual-functional colorimetric and fluorescent peptide-based probe for sequential detection of Cu^{2+} and S^{2-} in 100% aqueous buffered

solutions and living cells. *J. Hazard. Mater.* **2021**, *407*, 124388.

[10] Lei, C.; Qi, W.J.; Du, C.P.; Wang, Y.; Liu, C.; Huang, X.M.; Chang, X.J. A novel copper ion sensing fluorescent probe for fast detection of pyrophosphate and alkaline phosphatase. *New J. Chem.* **2021**, *45*, 3727-3734.

[21] Pan, J.; Yu, J.M.; Qiu, S.Y.; Zhu, A.Y.; Liu, Y.; Ban, X.X.; Li, W.; Yu, H.; Li, L.T. A novel di-benzimidazole-based fluorescent probe with high sensitivity and selectivity for copper ions. *J. Photochem. Photobiol., A* **2021**, *406*, 113018.

[22] Song, Y.T.; Tao, J.Y.; Wang, Y.; Cai, Z.C.; Fang, X.Y.; Wang, S.F.; Xu, H.J. A novel dual-responsive fluorescent probe for the detection of copper(II) and nickel(II) based on BODIPY derivatives. *Inorg. Chim. Acta* **2021**, *516*, 120099.

[23] Yang, Q.; Deng, S.; Jin, L.Y.; Jiang, Y.L.; Jin, C.; Wang, B.X.; Shen, J. A novel mitochondria-targeted fluorescent probe based on carbon dots for Cu^{2+} imaging in living cells and zebrafish. *J. Photochem. Photobiol., A* **2021**, *409*, 113143.

[24] Zhou, Z.L.; Tang, H.H.; Chen, S.Y.; Huang, Y.H.; Zhu, X.H.; Li, H.T.; Zhang, Y.Y.; Yao, S.Z. A turn-on red-emitting fluorescent probe for determination of copper(II) ions in food samples and living zebrafish. *Food Chem.* **2021**, *343*, 128513.

[25] Xu, P.P.; Liu, X.N.; Liu, L.; Zhu, W.J.; Li, C.; Fang, M. Carbazole-based colorimetric and fluorescent probe for Cu(II)and its utility in bio-imaging and real water samples. *J. Chin. Chem. Soc.* **2021**, *68*, 106-113.

[26] Khan, S.A.; Ullah, Q.; Parveen, H.; Mukhtar, S.; Alzahrani, K.A.; Asad, M. Synthesis and photo-physical investigation of novel imidazole derivative an efficient multimodal chemosensor for Cu(II) and fluoride ions. *J. Photochem. Photobiol., A* **2021**, *406*, 113022.

[27] Rajasekaran, D.; Venkatachalam, K.; Periasamy, V. "On-off-on" pyrene-based fluorescent chemo-sensor for the selective recognition of Cu^{2+} and S^{2-} ions and its utilization in live cell imaging. *Appl. Organomet. Chem.* **2020**, *43*, 5342.

[28] Wang, Y.; Hao, X.H.; Liang, L.X.; Gao, L.Y.; Ren, X.M.; Wu, Y.G.; Zhao, H.C. A coumarin-containing Schiff base fluorescent probe with AIE effect for the copper(ii) ion. *RSC Adv.* **2020**, *10*, 6109-6113.

[29] Dong, M.; Tang, J.Y.; Lv, Y.F.; Liu, Y.T.; Wang, J.F.; Wang, T.T.; Bian, J.Y.; Li, C.J. A dual-function

fluorescent probe for Hg(II) and Cu(II) ions with two mutually independent sensing pathways and its logic gate behavior. *Spectrochim. Acta, Part A* **2020**, *226*, 117645.

[30] Wang, Y.; Zhou, J.T.; Zhao, L.; Xu, B.C. A dual-responsive and highly sensitive fluorescent probe for Cu^{2+} and pH based on a dansyl derivative. *Dyes Pigm.* **2020**, *180*, 108513.

[31] Zhang, D.; Wang, Z.M.; Yang, J.; Yi, L.; Liao, L.F.; Xiao, X.L. Development of a method for the detection of Cu^{2+} in the environment and live cells using a synthesized spider web-like fluorescent probe. *Biosens. Bioelectron.* **2021**, *182*, 113174.

[32] Sonkar, A.K.; Rai, A.; Tripathi, K.; Yadav, R.; Shukla, M.; Chauhan, B.S.; Srikrishna, S.; Mishra, L. A dual optical probe with larger stokes shift for simultaneous detection of Cu^{2+} and Zn^{2+} ions and aggregation induced enhanced emission empowering selective detection of Cu^{2+} ions. *Sens. Actuators, B* **2021**, *327*, 129011.

[39] Shabunina, O.V.; Starnovskaya, E.S.; Shaitz, Y.K.; Kopchuk, D.S.; Sadieva, L.K.; Kim, G.A.; Taniya, O.S.; Nikonov, I.L.; Santra, S.; Zyryanov, G.V.; Charushin, V.N. A symmetrically substituted 5,5″-diaryl-2,2′,6′,2″-terpyridines as efficient fluorescence “turn-on” probes for Zn^{2+} in food/cosmetic samples and human urine. *J. Photochem. Photobiol., A* **2021**, *408*, 113101.

[33] Sun, J.; Li, T.R.; Liu, C.; Xue, J.; Tian, L.M.; Liu, K.; Li, S.L.; Yang, Z.Y. A dual probe for selective sensing of Zn (II) by fluorescent and Cu (II) by colorimetric methods in different systems based on 7,8-benzochromone-3-carbaldehyde -(fluorescein)hydrazone. *J. Photochem. Photobiol., A* **2021**, *406*, 113007.

[34] Kaur, N.; Kaur, B. Anthracene possessing amide functionality as a turn-on fluorescent probe for Cu^{2+} and Zn^{2+} ions. *J. Coord. Chem.* **2021**, *74*, 575-583.

[35] Erdemir, S.; Malkondu, S. Dual-channel responsive fluorescent sensor for the logic-controlled detection and bioimaging of Zn^{2+} and Hg^{2+}. *J. Mol. Liq.* **2021**, *326*, 115279.

[36] Li, W.Y.; Liu, Z.C.; Fang, B.Q.; Jin, M.; Tian, Y. Two-photon fluorescent Zn^{2+} probe for ratiometric imaging and biosensing of Zn^{2+} in living cells and larval zebrafish. *Biosens. Bioelectron.* **2020**, *148*, 111666.

[37] Fang, H.B.; Geng, S.S.; Hao, M.G.; Chen, Q.X.; Liu, M.L.; Liu, C.Y.; Tian, Z.Q.; Wang, C.J.; Takebe, T.; Guan, J.L.; Chen, Y.C.; Guo, Z.J.; He, W.J.; Diao, J.J. Simultaneous Zn^{2+} tracking in multiple organelles using super-resolution morphology-correlated organelle identification in living

cells. *Nat. Commun.* **2021**, *12*, 109.

[38] Muthusamy, S.; Zhu, D.W.; Rajalakshmi, K.; Zhu, W.H.; Wang, S.J.; Lee, K.B.; Zhao, L. Successive detection of zinc ion and citrate using a schiff base chemosensor for enhanced prostate cancer diagnosis in biosystems. *ACS Appl. Bio Mater.* **2021**, *4*, 1932-1941.

[39] Mohamad, N.S.; Zakaria, N.H.; Daud, N.; Tan, L.L.; Ta, G.C.; Heng, L.Y.; Hassan, N.I. The role of 8-amidoquinoline derivatives as fluorescent probes for zinc ion determination. *Sensors.* **2021**, *21*, 311.

[40] Li, N.N.; Bi, C.F.; Zhang, X.; Xu, C.G.; Fan, C.B.; Gao, W.S.; Zong, Z.A.; Zu, S.S.; Niu, C.F.; Fan, Y.H. A bifunctional probe based on naphthalene derivative for absorbance-ratiometic detection of Ag^{+} and fluorescence "turn-on" sensing of Zn^{2+} and its practical application in water samples, walnut and living cells. *J. Photochem. Photobiol., A* **2020**, *390*, 112299.

[41] Rajasekaran, D.; Venkatachalam, K.; Periasamy, V. A bisphenol based fluorescence chemosensor for the selective detection of Zn^{2+} and PPi ions and its bioluminescence imaging. *Spectrochim. Acta, Part A* **2020**, *242*, 118730.

[42] Fu, J.X.; Chang, Y.X.; Li, B.; Wang, X.H.; Xie, X.M.; Xu, K.X. A dual fluorescence probe for Zn^{2+} and Al^{3+} through differentially response and bioimaging in living cells. *Spectrochim. Acta, Part A* **2020**, *225*, 117493.

[43] Shang, Y.F.; Wang, H.L.; Bai, H. A coumarin-based turn-on chemosensor for selective detection of Zn(II) and application in live cell imaging. *Spectrochim. Acta, Part A* **2020**, *228*, 117746.

[44] Chen, S.S.; Sun, T.T.; Xie, Z.G.; Dong, D.W.; Zhang, N. A fluorescent sensor for intracellular Zn^{2+} based on cylindrical molecular brushes of poly(2-oxazoline) through ion-induced emission. *Polym. Chem.* **2020**, *11*, 6650-6657.

[45] Nguyen, M.H.; Nguyen. T.N.; Do, D.Q.; Nguyen, H.H.; Phung, Q.M.; Thirumalaivasan, N.; Wu, S.P.; Dinh, T.H. A highly selective fluorescent anthracene-based chemosensor for imaging Zn^{2+} in living cells and zebrafish. *Inorg. Chem. Commun.* **2020**, *115*, 107882.

[46] Hu, Y. Du, L.C.; Yang, J. A highly sensitive and selective chemosensors for detection of $Zn^{2+}$and its application in live cell imaging. *Inorg. Chim. Acta* **2020**, *509*, 119675.

[47] Wang, Y.Y.; Duan, H.N.; Shi, H.Y.; Zhang, S.W.; Xu, Y.F.; Zhu, W.P.; Qian, X.H. A highly sensitive fluorescent probe for tracking intracellular zinc ions and direct imaging of prostatic tissue in mice.

Chin. Chem. Lett **2020**, *31*, 2933-2936.

[48] Musib, D.; Devi, L.R.; Raza, M.K.; Chanu, S.B.; Roy, M. A new thiophene-based aggregation-induced emission chemosensor for selective detection of Zn^{2+} ions and its turn off. *Chem. Lett.* **2020**, *49*, 473-476.

[49] Liu, T.T.; Xu, J.; Liu, C.G.; Zeng, S.; Xing, Z.Y.; Sun, X.J.; Li, J.L. A novel dual-function probe for recognition and differentiation of Zn^{2+} and Al^{3+} and its application. *J. Mol. Liq.* **2020**, *300*, 112250.
